# intEgrating Smoking Cessation treatment As part of usual Psychological care for dEpression and anxiety (ESCAPE): A randomised and controlled, multi‐centre, acceptability and feasibility trial with nested qualitative methods

**DOI:** 10.1111/add.16718

**Published:** 2025-03-11

**Authors:** Gemma M. J. Taylor, Katherine Sawyer, Pamela Jacobsen, Tom P. Freeman, Anna Blackwell, Shadi Daryan, Chris Metcalfe, David Kessler, Marcus R. Munafò, Paul Aveyard

**Affiliations:** ^1^ Addiction and Mental Health Group, Department of Psychology University of Bath Bath UK; ^2^ NIHR Oxford Biomedical Research Centre John Radcliffe Hospital Oxford UK; ^3^ Population Health Sciences, Bristol Medical School University of Bristol Bristol UK; ^4^ Bath Centre for Mindfulness and Compassion, Department of Psychology University of Bath Bath UK; ^5^ Bristol Trials Centre, Bristol Medical School: Population Health Sciences University of Bristol Bristol UK; ^6^ Centre for Academic Primary Care, Bristol Medical School, Department of Population Health Sciences University of Bristol Bristol UK; ^7^ School of Psychological Science University of Bristol Bristol UK; ^8^ NIHR Bristol Biomedical Research Centre Bristol UK; ^9^ MRC Integrative Epidemiology Unit Bristol UK; ^10^ NIHR Oxford Health Biomedical Research Centre Warneford Hospital Oxford UK; ^11^ Nuffield Department of Primary Care Health Sciences University of Oxford Oxford UK; ^12^ NIHR Oxford and Thames Valley Applied Research Collaboration Oxford UK

**Keywords:** Anxiety, behavioural intervention, depression, IAPT, randomised controlled trial, smoking cessation, smoking treatment, talking therapies

## Abstract

**Background and aim:**

There is evidence that smoking cessation may improve depression and anxiety symptoms. We assessed the feasibility of implementing and trialling a smoking cessation intervention in services providing cognitive behavioural therapy (CBT) for common mental illness.

**Design, setting and participants:**

This study was a pragmatic, two‐armed, randomised, multi‐centre, acceptability and feasibility trial of a co‐designed smoking cessation intervention (ISRCTN99531779) involving United Kingdom National Health Service (NHS)‐funded services treating depression or anxiety among four NHS Trusts. Participants comprised adult daily smokers starting CBT for depression or anxiety [mean age 35.6 years, standard deviation (SD) = 12.7, 89.6% white] who smoked 14.3 (SD = 8.2) cigarettes/day with mean Generalised Anxiety Disorder Questionnaire‐7 (GAD‐7) and Patient Health Questionnaire‐9 (PHQ‐9) scores of 13.1 (SD = 4.9) and 14.5 (SD = 6.0). Sixty‐eight participants were allocated to the treatment group and 67 to control.

**Intervention and control:**

Both groups received CBT for depression or anxiety. The treatment group also received up to 12 sessions of integrated smoking cessation support. The control group was signposted to smoking cessation services post‐treatment.

**Measurements:**

Follow‐up was at 3 and 6 months. The primary outcome was ‘study completion’ by 3 months. Other outcomes included acceptability, satisfaction, feasibility, data completeness and mental health.

**Findings:**

At 3 months, treatment did not affect study completion [odds ratio (OR) = 0.81, 95% confidence interval (CI) = 0.31 to 2.09], did not harm mental health (PHQ‐9 difference: coefficient 0.01, 95% CI = −2.19 to 2.22); GAD‐7: coefficient 0.65, 95% CI = −1.59 to 2.90), but increased abstinence rates (OR = 8.69, 95% CI = 1.11 to 396.26). Recruitment was acceptable and key stakeholders were satisfied with the intervention.

**Conclusions:**

Among UK adult smokers receiving CBT treatment for depression or anxiety, a smoking cessation intervention within the CBT treatment was well received, did not interfere with the primary treatment goals and increased smoking cessation.

‘*I was signed off work with depression. I was asked if I smoked and I was encouraged not to try and quit… I was given anti‐depressants instead*’.

Smoker with depression, male, aged 37.

## INTRODUCTION

Smoking is the world's leading cause of preventable illness and death. One in every two people who smoke will die of a smoking‐related disease, unless they quit [1, 2]. The prevalence of smoking has decreased markedly from the 1970s in high‐income countries; for example, in the United Kingdom it has fallen from 46% to approximately 15% in 2023 [2, 3], and is similar in other high‐income countries [4].

However, smoking rates are higher in people with mental illness. People with depression or anxiety are twice as likely to smoke than those without. In the United Kingdom, the latest estimates from 2015 show that approximately 32% of people with depression or anxiety are smokers [5, 6]. Similar patterns are observed in other high‐income countries, such as the United States, where smoking prevalence is estimated at 30% for people with anxiety, and 36% for those with depression [4]. Despite being equally motivated to quit as individuals without mental illness [7], the odds of achieving abstinence after a quit attempt are 19% lower in people with mental illness compared with those without [8]. People with depression and anxiety are twice as likely to die from cancer [rate ratio = 1.92, 95% confidence interval (CI) = 1.91 to 1.94] [9] and cardiovascular disease (hazard ratio = 1.85, 95% CI = 1.53 to 2.24) [10].

Some evidence suggests that smoking may cause mental illness, and that stopping smoking improves mental health [11, 12]. The theory that quitting smoking can improve mental health is supported by plausible biological pathways, including the reversal of harm caused by smoking to neurotransmitter and inflammatory pathways [12]. Qualitative studies suggest that, although people with mental illness report that smoking improves their mental wellbeing, they accept that smoking may harm, and quitting might benefit mental health when evidence is explained [13]. This suggests that framing cessation as a treatment for mental illness could motivate quitting smoking.

In the United Kingdom, people with depression and/or anxiety have access to psychological therapy services, known as ‘Improving Access to Psychological Therapies’ (IAPT) (renamed ‘Talking Therapies’ in 2023), in which service users receive evidence‐based therapies to treat depression and anxiety (e.g. CBT). NHS IAPT services receive more than 1.5 million referrals a year [14] and could offer smoking cessation treatment, but it currently does not. Integrating smoking cessation support within psychological services has the potential to improve the physical and psychological health of service‐users. Health economic models based on US data indicate that integrating smoking cessation treatment into usual psychological care could prevent up to 125 000 deaths in the next 80 years in the United States alone [15].

Acknowledging the urgent public health need to reduce smoking prevalence in people with mental illness through targeted interventions [16], we conducted a pilot and feasibility study. In this study we integrated a co‐designed smoking cessation treatment into routine national health‐care psychological services for people with depression and anxiety. Although improvements in mental and physical health could be a benefit of such an intervention, prior to formally assessing such clinical outcomes we must first assess if implementing a smoking cessation intervention and conducting a trial of the intervention in IAPT settings is possible, and if offering smoking cessation in IAPT negatively impacts upon usual care.

Therefore, in this pilot and feasibility trial we aimed to: (1) establish the feasibility and acceptability of delivering a smoking cessation treatment together with IAPT usual care in terms of impact on study and IAPT completion, participant and practitioner acceptability and impact of the intervention on preliminary clinical outcomes, and (2) establish the feasibility of a multi‐centre randomised trial to compare the combined smoking cessation and IAPT treatment to usual IAPT treatment alone in terms of recruitment, data collection, randomisation and other trial procedures.

## METHODS

We pre‐registered and published the trial protocol and a detailed analysis plan (ISRCTN99531779) [17, 18]. Cleaning and analytical code is available via GitHub (https://github.com/gmjtaylor/escapepilotrct), and anonymised data are available via request to the University of Bath's Research Data Archive (https://researchdata.bath.ac.uk/).

### Study design

ESCAPE (intEgrating smoking cessation treatment as part of usual psychological care for dEpression and anxiety) is a pragmatic, two‐armed, randomised and controlled, multi‐centre, acceptability and feasibility trial with nested qualitative methods (ISRCTN99531779). Participants were recruited between 1 June 2018 and 31 August 2021 from four NHS Trust regions (five individual services). Both groups received standard IAPT psychological care (CBT) for their depression and anxiety symptoms. The treatment group additionally received integrated behavioural and pharmacological support for smoking cessation, delivered over up to 12 sessions. The control group received only usual IAPT care, with signposting to local smoking cessation services at the end of treatment. See Table S1 for the Consolidated Standards of Reporting Trials (CONSORT) checklist and Table S2 for schedule of enrolment, interventions and assessments.

### Participants

#### Inclusion/exclusion criteria

We included participants who were aged ≥ 18 years and currently diagnosed with depression and/or anxiety [Patient Health Questionnaire‐9 (PHQ‐9) score of ≥ 10 and/or Generalised Anxiety Disorder Questionnaire‐7 (GAD‐7) score of ≥ 8]. Other mental health comorbidities deemed treatable within IAPT were permitted. Participants were included if they reported smoking daily for ≥ 1 year and were interested in receiving help to quit smoking.

Participants were recruited before they were about to start IAPT treatment and were excluded if they had already started IAPT treatment, considered too unwell by IAPT practitioners to engage with the smoking cessation treatment or were pregnant or breastfeeding.

#### Setting

The trial was conducted among four English NHS Trusts and included five NHS IAPT services in the Midlands, London and Southwest of England. Participants were recruited among four NHS Trust regions from IAPT services providing CBT: Avon and Wiltshire Partnership NHS Trust (AWP, *n* = 25), Oxford Health NHS Foundation Trust (OX, *n* = 79), Northeast London Foundation NHS Trust (NEFLT, *n* = 21) and Dudley and Walsall Partnership NHS Trust (DW, *n* = 10).

#### Identification and consent

We tested two different recruitment models, IAPT practitioner‐led (June–December 2018) and academic‐led (January 2019–August 2021) (Figure S1 and Figure S2). Participants’ smoking status was assessed during service eligibility assessment and anyone smoking was asked whether they were interested in taking part in ‘a study about smoking and wellbeing’. It is standard to ask about smoking status in IAPT, but it is not regularly implemented, so we encouraged practitioners to ask about smoking to support recruitment. Contact details were passed to the NHS research team of the Trusts involved to assess final eligibility and gain informed consent over the telephone.

### Interventions

Participants received usual IAPT care in both intervention and control arms, which is CBT typically lasting 30–45 minutes per session delivered during one to 12 sessions (via the telephone or in person), according to a treatment plan agreed between the participant and practitioner.

#### Treatment: usual IAPT care plus smoking cessation treatment

The smoking cessation treatment programme was co‐designed by people with experience of depression and anxiety treatment who smoked and IAPT practitioners, and incorporated evidence‐based behavioural and medicinal smoking cessation interventions [19, 20]. The development is described elsewhere [13, 18, 21].

Behavioural support for smoking cessation was delivered in every CBT session and we asked practitioners to spend 5–15 minutes dedicated to smoking cessation treatment. Participants’ smoking cessation treatment was nested into the usual IAPT appointment schedule, which can last up to 12 sessions, but is decided on an individual basis depending on severity of illness and therapeutic requirements. We trained 51 non‐smoking IAPT practitioners as ‘level 2 smoking cessation advisers’. Training involved a level 1 online course as per National Centre for Smoking Cessation Training (NCSCT), followed by a level 2 in‐person 1‐day course provided by NCSCT [22]. We then provided monthly check‐in sessions to discuss delivery challenges. The full intervention manual is available in the Supporting information Appendix, and we have published a detailed practical guide elsewhere [21]. Practitioners used a smoking cessation intervention checklist and manual with examples to guide behavioural treatment for smoking cessation (Box 1). Behavioural smoking cessation support was based on NCSCT standard behavioural treatment and involved co‐designed psychoeducation about the evidence that cessation reduced the severity of depression and anxiety [11, 23]. Participants were encouraged to set a quit day on their second appointment (Box 1). Psychoeducation was delivered using a co‐designed CBT framework to help patients understand the vicious cycle of tobacco withdrawal worsening mental wellbeing that is temporarily relieved, but ultimately maintained by continued smoking. This explains that tobacco withdrawal symptoms of adverse mood are relieved by smoking, yet at the same time caused by smoking, and therefore why cessation can improve mental health [11, 23].

BOX 1 **Overview of intervention components.^a^**

**Table:** 

IAPT treatment appointment	1	2	3–5	6
Smoking cessation treatment session	Pre‐quit	Quit day	Follow‐up	Final
Minutes	10–15	10–15	5–10	5–10
Address beliefs about smoking and mental health	1✔️	✔️	✔️	✔️
Inform the client about the treatment programme	✔️			
Assess current smoking	✔️			
Assess past quit attempts	✔️			
Explain how tobacco dependence develops and assess nicotine dependence	✔️			
Explain and conduct carbon monoxide (CO) monitoring (not available via telephone)	✔️	✔️	✔️	✔️
Explain the importance of abrupt cessation and the ‘not a puff’ rule	✔️	✔️	✔️	✔️
Inform the client about withdrawal symptoms	✔️			
Discuss stop smoking medications/aids	✔️			
Provide client NRT/vouchers or refer to pharmacy/GP for varenicline	✔️	✔️	✔️	✔️
Provide client ‘participant information pack’ (email, handout or post)	✔️	✔️	✔️	✔️
Set the ‘Quit Date’	✔️			
Prompt a commitment from the client	✔️	✔️		
Check on client's progress			✔️	✔️
Confirm readiness and ability to quit		✔️		
Confirm that the client has a sufficient supply of smoking cessation medication/aids		✔️	✔️	✔️
Advise about continued medication use and ensure that the client knows where to obtain further supplies				✔️
Enquire about medication use			✔️	✔️
Discuss withdrawal symptoms and cravings, and how to cope		✔️	✔️	
Advise on changing routine		✔️		
Discuss how to address the issue of the client's smoking contacts and how the client can get support during their quit attempt		✔️		
Discuss any difficult situations experienced and methods of coping			✔️	✔️
Address any potential high‐risk situations in the coming week		✔️	✔️	
Discuss plans and provide a summary	✔️	✔️	✔️	✔️

^a^Sources: National Centre for Smoking Cessation and Training [22]; Taylor *et al*. [18, 23].

Participants were encouraged to use any smoking cessation medicine or aid to help them to quit and were not randomised to a specific product. The practitioner arranged for participants to be prescribed smoking cessation medication (combined nicotine replacement therapy (NRT) or varenicline) or recommend purchase of e‐cigarettes. In one Trust participants received medication without charge, and in others NHS prescription fees were levied for people who were not exempt. E‐cigarettes were purchased by participants.

#### Control: usual IAPT care plus delayed signposting to local smoking cessation service

Participants received usual IAPT care plus signposting to their local smoking cessation service during their final IAPT appointment (which may or may not have been attended). The goal of usual care was to improve depression and anxiety symptoms.

### Randomisation

The randomisation sequence was generated using Stata software by a statistician not involved in recruitment to the study (C.M.). Random allocation was 1:1 between the two study groups and was stratified by Trust, with random permuted blocks. Allocation was concealed until after enrolment, as researchers had to enter participants’ details into the study's REDCAP database [24] before allocation was revealed. Researchers informed the clinical team of allocation.

### Blinding

Due to the nature of the intervention, it was not possible to blind participants and practitioners delivering the intervention to allocation. Outcome assessments were conducted blind to allocation. Independent researchers collected follow‐up data from patients and IAPT patient management systems blinded to treatment allocation.

### Data collection

We tested two data collection models, NHS‐led, whereby NHS staff were responsible for collecting data and academic‐led where academic research staff were responsible for collecting data. Data collection was monitored monthly. Follow‐up was conducted 3 and 6 months after the first IAPT appointment. We collected data from patients over the telephone or in person. Where follow‐up directly with patients was not possible, we extracted appointment data, PHQ‐9, GAD‐7 and smoking status from IAPT patient management systems within a 2‐week window around participant follow‐up dates. Participants were given a £10 shopping voucher for completing both follow‐ups.

### Outcomes

#### Acceptability and feasibility outcomes

Our primary feasibility outcome assessed whether offering integrated smoking cessation support impacted upon study completion [17, 18]. Participants were considered a completer if they completed IAPT care, achieved abstinence or made a quit attempt. We also assessed the following.

- Recruitment rate.
- Participant acceptability and satisfaction of smoking cessation treatment using the modified version of the ‘Stop Smoking Service Client Satisfaction Survey’ [22].
- Practitioner acceptability and satisfaction with the smoking cessation treatment using: ‘Clinician Self‐Report Intervention Acceptability Questionnaire’, the ‘Acceptability of Intervention Measure’, ‘Intervention Appropriateness Measure’, ‘Feasibility of Intervention Measure’ and ‘Sustainability of Intervention Measure’ [25].
- Service‐related feasibility outcomes: the number of planned, completed and missed IAPT appointments, IAPT treatment status (active/discontinued/completed/discharged).
- Smoking cessation treatment‐related feasibility outcomes: completion of smoking cessation sessions during IAPT appointments, retention in smoking cessation treatment, average duration of smoking cessation treatment sessions in minutes, patient use of smoking cessation medication (type of medicine and dose and e‐cigarette use).
- Blinded outcome data collection: we asked researchers: ‘At the very start of the telephone call—are you aware of which treatment arm the participant was allocated to?’ (yes/no).
- Data collection and follow‐up rates.

#### Clinical outcomes

We collected data on the probable primary and secondary outcomes in a future effectiveness trial.

- Point‐prevalence, self‐reported 7‐day smoking abstinence with biological‐verification [exhaled carbon monoxide concentration ≤ 10 parts per million (p.p.m.), or a cotinine level ≤ 10 nanograms per millilitre of saliva] at 3 and 6 months follow‐up.
- PHQ‐9, GAD‐7, Heaviness of Smoking Index (HSI), number of cigarettes per day (CPD) and adverse events.

### Sample size calculation

As pre‐specified in our original published protocol [17], we calculated our sample size as follows. Assuming a sample size of 100 participants at follow‐up, we used a standard formula to calculate the binomial exact confidence interval (CI) [26, 27]. Based on this formula the study had sufficient precision to estimate that 40% or more participants would continue with treatment in the intervention arm to within a 95% CI of 26 to 55%. We assumed that 36% of participants would be lost to follow‐up [28], and therefore the study required a sample size of 157 at baseline.

### Statistical analysis

All analyses were conducted using Stata version 17 MP following a pre‐registered analysis plan [18]. Cleaning and analytical code is available via GitHub https://github.com/gmjtaylor/escapepilotrct.git.

For the primary feasibility outcome, we presented the proportion of ‘study completers’ in each arm and as calculated by *n* study completers in arm/*n* randomised to arm at baseline, and used exact logistic regression models to estimate the odds ratio (OR) and 95% CI for the effect of allocation to treatment or control, on study completion at 3 and 6 months follow‐up. For randomised controlled trial (RCT) effectiveness outcomes (i.e. smoking cessation, GAD‐7, PHQ‐9, CPD and HSI), we reported data completeness at each follow‐up and the number of values that were extracted from IAPT patient management systems where patients were lost‐to‐follow‐up or where data were missing. Those with missing data about study completion were assumed to be a ‘non‐completer’.

For dichotomous outcomes, we present numerators, denominators and percentages by trial arm at 3 and 6 months follow‐up. We conducted exact logistic regression models to produce ORs and 95% CIs for the effect of allocation to treatment arm on outcomes at 3 and 6 months follow‐up. Where smoking cessation values were missing, we assumed that those with missing values continued smoking or had relapsed to smoking as recommended by West *et al*. [29].

For continuous outcomes we present means and standard deviations (SD) by trial arm at 3 and 6 months follow‐up. We conducted linear regression models to produce beta coefficients for the effect of allocation to treatment arm on outcomes at 3 and 6 months follow‐up with adjustment for baseline outcome values. In our detailed pre‐registered analysis plan (https://osf.io/kq9te) we specified that we would conduct both complete case and imputed analyses. The goal was to present the analysis of observed data as the primary estimates and explore the potential effects of missing data through sensitivity analyses with different assumptions. Therefore, where continuous outcome data were missing, we used multivariate multiple imputation under the assumption that data were missing at random [30, 31]. We repeated descriptive and inferential analyses using complete case and imputed missing data and using regression models.

We chose not to adjust comparisons for potentially prognostic covariates because, given that this is a feasibility study, the data probably lack the precision needed to accurately estimate the effects of the intervention. Therefore, we used a simpler and more straightforward approach to the analysis wherever possible.

### Qualitative analysis

For qualitative analyses methods are reported in detail elsewhere [18, 32, 33]. In summary, we conducted individual interviews with IAPT practitioners to explore their perceptions of the acceptability of the smoking cessation treatment and data collection procedures, positive and negative impacts of smoking cessation treatment on IAPT usual care and mental health recovery. Interviews with participants explored perceptions of IAPT practitioner ability to deliver the smoking cessation treatment, acceptability of the smoking cessation treatment, positive and negative impacts of smoking cessation treatment on IAPT usual care and mental health recovery and data collection procedures. Qualitative data were analysed using a thematic approach, following guidance outlined by Braun & Clarke [34]; thematic analysis allowed for both anticipated (i.e. deductive coding) and emergent themes (i.e. inductive coding). All data were anonymised and to ensure the quality of data transcription a researcher completed a 50% check of audio data against the transcripts. NVivo software was used to code and apply the analytical framework.

### Protocol amendments and deviations

We made 22 protocol amendments during the study period, details are available via the Open Science Framework (OSF) (https://osf.io/kq9te/) (Table S3). We aimed to collect and analyse recordings of intervention delivery; however, IAPT practitioners and management did not have the capacity to collect recordings.

The primary purpose of feasibility studies is to generate findings that help to determine whether an intervention should be recommended for efficacy testing. During the study, we made the decision to modify our primary feasibility outcome from the original ISCRTN registration to the published study protocol [37] to ensure that it aligned with the perspectives and needs of prospective commissioners and intervention providers. The original ISCRTN outcome was ‘Retention in the smoking cessation treatment, measured at treatment appointments 1 to 6’. However, after consulting with IAPT commissioners and service and clinical managers, it became evident that the most crucial factor for them was whether offering a smoking cessation intervention would lead service users to disengage with the service, and we added this as a co‐primary outcome. We also stated in our published protocol that we would use random‐effects models adding Trust as a random effect; however, there was no expectation that intervention delivery would differ between Trust, therefore we decided not to adjust for Trust.

We wrote our justification on 1 August 2021 and decided to stop recruitment 31 August 2021 (https://osf.io/kq9te/). For our complete case precision estimate, at the time of deciding to end recruitment we had enrolled 128 participants in the trial, assuming 36% attrition [17, 18], therefore leaving us with 82 participants at follow‐up. We estimated that if 40% of remaining participants continued with smoking cessation treatment in the treatment arm, this would provide us a 95% CI of 24 to 55%. For our intention‐to‐treat precision estimate, if 25% (16 of 64) of remaining participants continued with smoking cessation treatment in the treatment arm, this would provide us a 95% CI of 24 to 55%.

## RESULTS

### Participants

**FIGURE 1:**
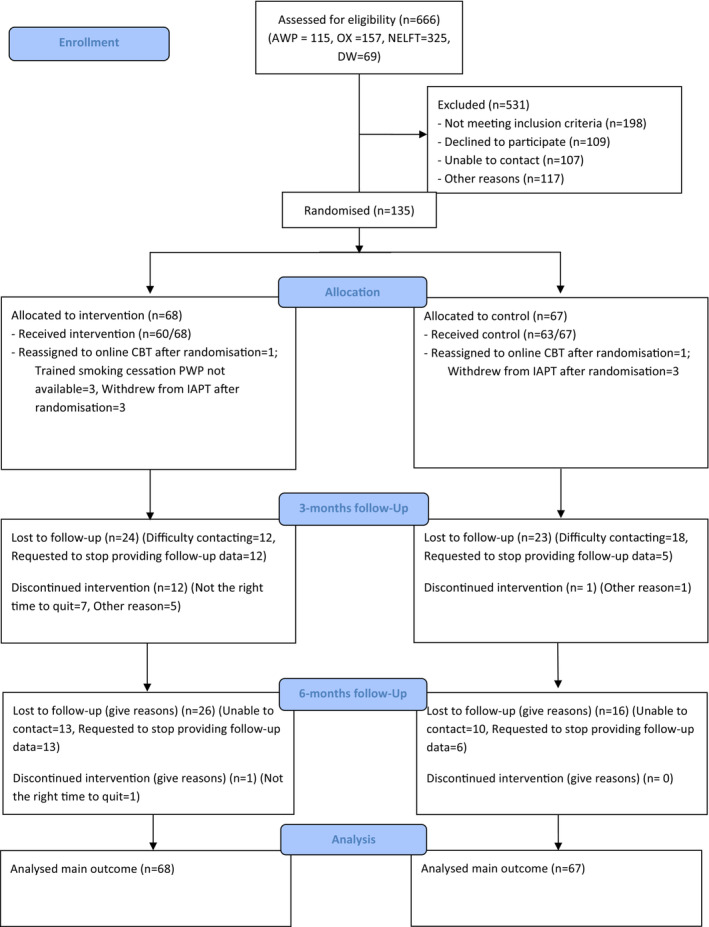
Consolidated Standards of Reporting Trials (CONSORT) flow diagram: numbers of participants who were assessed for eligibility, randomly assigned, received intended treatment and were assessed for each objective.

Sixty‐eight participants were assigned to usual care plus integrated smoking cessation intervention (treatment) and 67 were allocated to usual care (control). Figure 1 presents the CONSORT flow diagram. Participants’ characteristics were balanced across trial arms. Participants’ mean age was 35.6 (SD = 12.7), 89.6% (121 of 135) were White, they smoked 14.3 (SD = 8.2) CPD and had a median IMD score of 14.3 [interquartile range (IQR) = 8.5 to 25.8]. Participants had moderate severity depression and anxiety, with mean GAD‐7 and PHQ‐9 scores of 13.1 (SD = 4.9) and 14.5 (SD = 6.0) (Table 1). The population was representative of IAPT service users in sex, age and education and participants with common mental illness from other RCTs of smoking cessation interventions [35].

**TABLE 1:** Baseline and pre‐clinical characteristics.

		Control *n* = 67	Treatment *n* = 68
Trust, number (*n*) and percentage (%)	Avon and Wiltshire Mental Health Partnership NHS Trust	12 (17.9%)	13 (19.1%)
	Oxford Health NHS Foundation Trust	39 (58.2%)	40 (58.8%)
	Northeast London NHS Foundation Trust	12 (17.9%)	9 (13.2%)
	Black Country Healthcare NHS Foundation Trust	4 (6.0%)	6 (8.8%)
Mean age in years (SD)		33.7 (11.9)	37.4 (13.3)
Gender, *n* (%)	Male	27 (40.3%)	22 (32.4%)
	Female	40 (59.7%)	46 (67.6%)
Ethnicity, *n* (%)	White	60 (89.6%)	61 (89.7%)
	Mixed	4 (6.0%)	3 (4.4%)
	Indian	1 (1.5%)	1 (1.5%)
	Pakistani	2 (3.0%)	1 (1.5%)
	Bangladeshi	0 (0.0%)	1 (1.5%)
	Other	0 (0.0%)	1 (1.5%)
Highest level of education, *n* (%)	Some high school	1 (1.6%)	1 (1.5%)
	GCSE/O‐grade/equivalent	15 (24.6%)	18 (26.9%)
	A‐level equivalent	6 (9.8%)	12 (17.9%)
	Apprenticeship	5 (8.2%)	3 (4.5%)
	Other vocational	13 (21.3%)	14 (20.9%)
	Degree	16 (26.2%)	14 (20.9%)
	Higher degree	5 (8.2%)	5 (7.5%)
Median IMD score (interquartile range)		13.3 (9.3 to 25.8)	14.8 (8.4 to 25.8)
Mean PHQ‐9 (SD)^a^		15.0 (5.9)	14.0 (6.1)
Mean GAD‐7 (SD)^a^		13.8 (4.7)	12.4 (5.1)
Comorbid anxiety, *n* (%)^a^	No	33 (49.3%)	42 (61.8%)
	Yes	34 (50.7%)	26 (38.2%)
Comorbid depression, *n* (%)^a^	No	12 (17.9%	16 (23.5%)
	Yes	55 (82.1%)	52 (76.5%)
Comorbid panic attacks, *n* (%)^a^	No	58 (86.6%)	60 (88.2%)
	Yes	9 (13.4%)	8 (11.8%)
Comorbid OCD, *n* (%)^a^	No	60 (89.6%)	62 (91.2%)
	Yes	7 (10.4%)	6 (8.8%)
Other comorbid mental health condition, *n* (%)^a^	No	54 (80.6%)	55 (80.9%)
	Yes	13 (19.4%)	13 (19.1%)
Mean HSI (SD)^a^		2.1 (1.6)	2.6 (1.6)
Mean CPD (SD)^a^		13.0 (7.8)	15.5 (8.4)
Mean number of previous quit attempts (SD)		5.0 (6.9)	3.7 (6.7)

Abbreviations: IMD = Index of Multiple Deprivation, PHQ‐9 = Patient Health Questionnaire‐9, GAD‐7 = Generalised Anxiety Disorder Questionnaire, OCD = obsessive–compulsive disorder, HSI = Heaviness of Smoking Index, CPD = cigarettes per day; SD = standard deviation.

^a^ Pre‐clinical characteristics.

### Acceptability and feasibility outcomes

#### Main outcome

By 3 months, there was no strong evidence that offering smoking cessation treatment affected IAPT completion rates by trial arm (Table 2), OR = 0.81, 95% CI = 0.31 to 2.09, *P* = 0.79. This result remained consistent when analysing both complete case and imputed data. The same results were observed at the 6 month follow‐up (Tables 2 and 3). Participants engaged with the smoking cessation intervention, indicated by higher quit attempts, and higher biologically‐verified smoking cessation rates in the intervention arm (Table 2). Results were similar for 6‐month follow‐up (Table 3).

**TABLE 2:** The effect of random allocation to intervention arm on main feasibility and clinical outcomes at 3 months. Data presented are odds ratios (OR) or beta‐coefficients and 95% confidence intervals (CI) derived from regression models. Estimates derived from complete case and imputed data are presented.^a^

Exact logistic regression models		Control	Treatment	Unadjusted OR (95% CI)
IAPT treatment completion status, *n* completed (%)	Complete case	14/48 (29.2%)	12/52 (23.1%)	0.73 (0.27 to 1.96), *P* = 0.64
	Imputed missing data	14/67 (20.9%)	12/68 (17.6%)	0.81 (0.31 to 2.09), *P* = 0.79
Biologically‐verified self‐reported 7‐day smoking abstinence, *n* quit (%)	Complete case	1/43 (2.3%)	8/45 (17.8%)	8.90 (1.11 to 412.32)
	Imputed missing data	1/67 (1.5%)	8/68 (11.8%)	8.69 (1.11 to 396.26)
Quit attempt, *n* attempted (%)	Complete case	4/44 (9.1%)	14/55 (25.1%)	3.38 (0.95 to 15.30)
	Imputed missing data	4/67 (6.0%)	14/68 (20.6%)	4.04 (1.18 to 17.88)

Linear regression models				Beta‐coefficient (95% CI)^b^
GAD‐7, mean (SD)	Complete case	9.0 (6.1), *n* = 48	9.5 (5.6), *n* = 44	0.54 (−1.75 to 2.83), *n* = 90
	Imputed missing data	9.7 (5.8), *n* = 67	9.7 (5.5), *n* = 68	0.65 (−1.59 to 2.90), *n* = 135
PHQ‐9, mean (SD)	Complete case	9.9 (6.2), *n* = 49	9.9 (5.8), *n* = 46	−0.27 (−2.39 to 1.85), *n* = 93
	Imputed missing data	10.4 (6.0), *n* = 67	10.0 (5.9), *n* = 68	0.01 (−2.19 to 2.22), *n* = 135
CPD, mean (SD)	Complete case	9.7 (5.8), *n* = 34	8.7 (8.6), *n* = 35	−1.97 (−5.08 to 1.13), *n* = 68
	Imputed missing data	9.4 (7.0), *n* = 67	9.5 (8.6), *n* = 68	−1.09 (−5.38 to 3.20), *n* = 135
HSI, mean (SD)	Complete case	1.7 (1.4), *n* = 37	1.7 (1.6), *n* = 35	−0.24 (−0.84 to 0.36), *n* = 71
	Imputed missing data	2.0 (1.5), *n* = 67	2.0 (1.7), *n* = 68	−0.17 (−0.94 to 0.60), *n* = 135

Abbreviations: CPD = cigarettes per day; GAD‐7 = Generalised Anxiety Disorder Questionnaire; HSI = Heaviness of Smoking Index; PHQ‐9 = Patient Health Questionnaire‐9. *P*‐values only reported for primary trial outcome.

^a^ See analysis section for missing value assumptions.

^b^ Models were adjusted for baseline outcome values.

**TABLE 3:** The effect of random allocation to intervention arm on main feasibility and clinical outcomes at 6 months. Data presented are odds ratios (OR) or beta‐coefficients and 95% confidence intervals (CI) derived from regression models. Estimates derived from complete case and imputed data are presented.^a^

Exact logistic regression models		Control	Treatment	Unadjusted OR (95% CI)
IAPT treatment completion status, *n* completed (%)	Complete case	22/54 (40.7%)	17/53 (32.1%)	0.69 (0.29 to 1.63), *P* = 0.47
	Imputed missing data	22/67 (32.8%)	17/68 (25.0%)	0.68 (0.30 to 1.53), *P* = 0.42
Biologically‐verified self‐reported 7‐day smoking abstinence, *n* quit (%)	Complete case	4/50 (8.0%)	10/40 (25.0%)	3.78 (0.98 to 18.03)
	Imputed missing data	4/67 (6.0%)	10/68 (14.7%)	2.70 (0.73 to 12.43)
Quit attempt, *n* attempted (%)	Complete case	9/52 (17.3%)	16/56 (28.6%)	1.90 (0.70 to 5.47)
	Imputed missing data	9/67 (13.4%)	16/68 (23.5%)	1.97 (0.75 to 5.53)

Linear regression models				Beta‐coefficient (95% CI)^b^
GAD‐7, mean (SD)	Complete case	8.9 (6.3), *n* = 50	8.2 (5.9), *n* = 40	0.04 (−2.26 to 2.33), *n* = 86
	Imputed missing data	9.7 (6.1), *n* = 67	8.4 (6.2), *n* = 68	0.10 (−2.13 to 2.33), *n* = 135
PHQ‐9, mean (SD)	Complete case	9.2 (6.6), *n* = 50	9.8 (6.9), *n* = 40	1.09 (−1.39 to 3.58), *n* = 86
	Imputed missing data	9.7 (6.7), *n* = 67	10.0 (7.2), *n* = 68	0.96 (−1.75 to 3.66), *n* = 135
CPD, mean (SD)	Complete case	9.7 (8.0), *n* = 43	9.3 (9.4), *n* = 35	−1.94 (−4.88 to 1.01), *n* = 74
	Imputed missing data	9.9 (8.5), *n* = 67	10.9 (9.6), *n* = 68	−1.64 (−5.11 to 1.83), *n* = 135
HSI, mean (SD)	Complete case	1.6 (1.5), *n* = 43	1.8 (1.8), N = 35	−0.19 (−0.73 to 0.35), *n* = 74
	Imputed missing data	2.0 (1.7), *n* = 67	2.2 (1.9), *n* = 68	−0.12 (−0.81 to 0.57), *n* = 135

Abbreviations: CPD = cigarettes per day; GAD‐7 = Generalised Anxiety Disorder Questionnaire; HSI = Heaviness of Smoking Index; PHQ‐9 = Patient Health Questionnaire‐9. *P*‐values only reported for primary trial outcome.

^a^ See analysis section for missing value assumptions.

^b^ Models were adjusted for baseline outcome values.

#### Recruitment

We recruited 135 of the planned 157 participants. Recruitment was slower than expected and interrupted by the COVID‐19 pandemic, so we ended recruitment early (see Supporting information, Appendix 1.3 for justification). The recruitment rate varied between Trusts (Figure S3). Our median recruitment rate per month, by Trust, was two participants.

#### Participant acceptability and satisfaction smoking cessation treatment

At 3 months follow‐up, 21 of 68 participants had completed the smoking cessation intervention. Overall, 90% (19 of 21) of participants reported that they were satisfied with the intervention (Table S4), and 76% (16 of 21) would recommend the treatment to others who want to stop smoking. Qualitative analyses of participant interview data have been published elsewhere [32, 33]. In summary, participants accepted the smoking cessation treatment offered in IAPT and felt that IAPT had a natural infrastructure for supporting integrated treatment. Some participants did not understand the tobacco withdrawal cycle.

#### IAPT practitioner acceptability and satisfaction with the smoking cessation treatment

We approached 51 practitioners to complete the survey and 18 responded. Most practitioners, 61% (11 of 18), were satisfied with the delivery of the smoking cessation intervention, and 83% (15 of 18) would be happy to offer the intervention again (Table S5). Practitioners expressed a keen interest in using CBT for addiction treatment within IAPT services, considering it valuable. They found that it was easy to integrate smoking cessation into structured CBT and perceived that participants’ mood improved on smoking cessation. Practitioners felt that the intervention was sometimes difficult to fit into session duration; however, they valued the intuitive training materials and reported that intervention delivery became second nature. Detailed themes and subthemes are presented in the Appendix (Table S6).

#### Service‐related feasibility outcomes

On average, the number of missed and completed IAPT appointments was similar in both trial arms at 3 and 6 months (Table 4).

**TABLE 4:** Service‐related feasibility outcomes. Number of missed, completed, and planned appointments between baseline and follow‐up. Data are presented as means [standard deviation (SD)] and percentage (%) by trial arm and by follow‐up.

	3‐month		6‐month	
	Control	Treatment	Control	Treatment
	*n* = 67	*n* = 68	*n* = 67	*n* = 68
Mean (SD) number of missed appointments	1.5 (1.7)	1.7 (1.7)	1.6 (1.8)	1.8 (1.7)
Mean (SD) number of completed appointments	3.9 (2.7)	4.0 (2.7)	4.2 (3.2)	4.1 (2.7)
Mean (SD) number of planned appointments	3.7 (2.9)	5.1 (2.8)	4.2 (3.0)	5.4 (2.5)

#### Smoking cessation treatment‐related feasibility outcomes

Seventy‐one per cent of IAPT appointments contained smoking cessation behavioural support. In the treatment arm, on average 3.7 (SD = 2.1) smoking sessions were delivered during IAPT treatment. Smoking cessation treatment assessment appointments lasted a mean of 17.0 (SD = 8.3) minutes and follow‐up sessions were 11.2 minutes (SD = 5.7) (Table S7). At the assessment session, smoking cessation medicine or aid recommendation was recorded by practitioners for 37 participants, and 65% (24 of 37) of participants were recommended dual‐form NRT. At the second quit day appointment, 42 participants were recorded as using smoking cessation medication or an aid, and 65% (28/42) of these were using dual‐form NRT (Tables S8 and S9).

#### Blinded outcome data collection

Where data were provided, seven of 45 (16%) researchers in the control arm and eight of 53 (15%) in the treatment arm were aware of participant allocation at the start of the follow‐up telephone call at 3 months and six of 45 (13%) and eight of 46 (17%) at 6 months. This rate did not appear to differ between trial arms (Table S12), and the majority of unblinding occurred in one Trust at 3 months and among two Trusts at 6 months (Tables S10 and S11).

#### Data collection

There were no differences between trial arms in the number of days between randomisation and first IAPT appointment, and first IAPT appointment and 3 and 6 months follow‐up (Table S12). During the study period, 28 researchers were involved in data collection. Data were more complete for periods where academic researchers supported collection, and monthly data completeness ranged from 0 to 100% (Tables S13–S15).

#### Follow‐up rates

We closely monitored follow‐up rates (Tables S16 and S17, Supporting information, Appendix 2.1.3 and 2.1.4). In December 2019 it was noted that follow‐up rates were low in Oxford and AWP, with rates between 46 and 62%. To address this, several measures were implemented, including researcher training, biweekly missing data reports and data imputation (as extracted from IAPT patient management systems), and we introduced out‐of‐hours follow‐ups, welcome and reminder letters, prompts on REDCAP and a study newsletter. Follow‐up rates improved by September 2020, although 6‐month follow‐up rates remained low. We changed responsibility for data collection from being NHS staff‐led to academic staff‐led in September 2021, which further improved follow‐up rates.

Withdrawal rates were higher in the treatment arm because participants in this arm were repeatedly asked if they wished to continue, unlike control participants. The protocol was amended in December 2020 (approved April 2021) to remove this question. The differential questioning probably caused higher dropout rates.

#### Clinical outcomes

At 3 months, 88 of 135 participants and at 6 months 90 of 135 participants provided smoking status data (Table 2). Assuming that those missing outcome data were still smoking, eight of 68 participants in the treatment and one of 67 in the control arm reported 7‐day abstinence and were biologically‐verified as abstinent, OR = 8.69 (95% CI = 1.11 to 396.26) (Table 2). At 6 months, 10 of 68 were abstinent in treatment and four of 67 in control, 2.70 (0.73 to 12.43) (Table 3). There were no reported adverse events during this study.

Using complete‐case data, at 3 months follow‐up mean scores and SDs for the GAD‐7 were 9.5 (5.6) in the treatment arm and 9.0 (6.1) in the control arm; PHQ‐9 were 9.9 (5.8) for treatment and 9.9 (6.2) for control; CPD were 8.7 (8.6) for treatment and 9.7 (5.8) for control; and HSI were 1.7 (1.6) for treatment; and 1.7 (1.4) for control. With missing data imputed, means and SDs were similar (Table 2). After imputing missing outcome values there was no evidence for an effect of allocation to treatment arm on anxiety symptoms, beta coefficient 0.65 (95% CI = −1.59 to 2.90); depression symptoms 0.01 (−2.19 to 2.22); CPD −1.09 (–5.38 to 3.20); or HSI = **−**0.17 (–0.94 to 0.60). Effect estimates derived from complete case data were comparable (Table 2).

Using complete‐case data, at 6 months follow‐up mean scores and SDs for the GAD‐7 were 8.2 (5.9) in the treatment arm and 8.9 (6.3) in the control arm, PHQ‐9 were 9.8 (6.9) for treatment and 9.2 (6.6) for control; CPD were 9.3 (9.4) for treatment and 9.7 (8.0) for control; and HSI were 1.8 (1.8) for treatment and 1.6 (1.5) for control. With missing data imputed, means and SDs were similar (Table 3). After imputing missing outcome values, there was no evidence for an effect of allocation to treatment arm on anxiety symptoms, beta coefficient 0.10 (95% CI = –2.13 to 2.33); depression symptoms 0.96 (–1.75 to 3.66); CPD −1.64 (–5.11 to 1.83); or HSI −0.12 (–0.81 to 0.57). Effect estimates derived from complete case data were comparable (Table 3).

In an exploratory analysis we examined change in GAD‐7 and PHQ‐9 scores from baseline to follow‐up in quitters and those who continued smoking, stratified by trial arm. Mean change scores indicated that among trial arms there was a greater improvement in GAD‐7 and PHQ‐9 at 3 months follow‐up and PHQ‐9 at 6 months follow‐up in those who had quit smoking (Table 5).

**TABLE 5:** Change in GAD‐7 and PHQ‐9 scores from baseline to 3‐ and 6‐months follow‐up for people who quit or who continued smoking in treatment and control arms. Mean change and standard deviations (SDs) derived from complete case data are presented.^a^

	3‐months		6‐months	
	Control	Treatment	Control	Treatment
GAD‐7, mean change (SD)				
Quit	−15.00 (0.00), *n* = 1	−6.38 (6.30), *n* = 8	−4.33 (9.29), *n* = 3	−5.90 (4.28), *n* = 10
Smoking	−5.05 (5.34), *n* = 40	−3.20 (5.67), *n* = 35	−4.93 (5.02), *n* = 44	−3.79 (5.71), *n* = 28
PHQ‐9, mean change (SD)				
Quit	−9.00 (0.00), *n* = 1	−6.50 (5.26), *n* = 8	−9.33 (5.13), *n* = 3	−5.20 (4.71), *n* = 10
Smoking	−5.17 (5.23), *n* = 41	−4.86 (5.89), *n* = 37	−6.09 (5.77), *n* = 44	−4.75 (6.93), *n* = 28

Abbreviations: PHQ‐9 = Patient Health Questionnaire‐9; GAD‐7 = Generalised Anxiety Disorder Questionnaire.

^a^ Negative number (−) indicates a reduction/improvement in symptoms.

## DISCUSSION

We found no strong evidence to suggest that offering smoking cessation treatment affected study completion or usual IAPT treatment, and we found evidence to suggest that participants engaged with the smoking cessation treatment. We recruited at an acceptable rate among four NHS Trusts, and attrition was typical of smoking cessation trials in this population. Practitioners and participants reported that they were satisfied with the intervention, and we found evidence that integrating smoking cessation treatment into may increase the rate of cessation in people with depression and/or anxiety. Those in the intervention arm achieved higher quit rates, with no evidence of worsening depression or anxiety symptoms compared to control. However, there are some feasibility concerns in running a full‐sized trial in terms of intervention implementation, given practitioner case‐load and retention and concerns about the rate of recruiting Trusts. A future trial would need to address these.

### Strengths

The trial methods, primary outcomes and analysis plan were peer‐reviewed, published and pre‐registered [17, 18]. In designing the trial and the intervention, we used co‐design to adapt and enhance an evidence‐based smoking cessation treatment package together with IAPT service users, staff and commissioners and smoking cessation advisers. The trial was adequately sized to estimate precisely if offering smoking cessation treatment impacted upon usual IAPT care. We recruited successfully from an under‐served population and integrated a smoking cessation treatment into routine IAPT care throughout four NHS Trusts. We also biologically verified self‐reported smoking cessation at follow‐ups, strengthening evidence of intervention promise. We improved overall follow‐up rates and reduced differential attrition through researcher retraining and implementing protocol changes. We could not recover the 6‐month follow‐up differential attrition, as the numbers had already been significantly impacted early in the trial. However, the lessons learned from this trial will be important for future trials.

### Limitations

We were unable to record IAPT appointments, meaning that it was not possible to fully assess intervention adherence or participant reaction to the treatment. Most participants consented to having their sessions recorded, but Trusts felt that recording the data was unethical, despite approval by NHS ethics committee for this. Researcher blinding to treatment allocation was unsuccessful in 13–16% of follow‐ups; most unblinding occurred at one site. This may have introduced bias in data collection; however, many variables were already collected as part of usual care via the IAPT system, such as whether a participant dropped out of IAPT, and abstinence was verified biologically. A slightly higher proportion of participants requested to stop providing follow‐up data, and who were difficult to contact in the intervention arm. We believe that withdrawal rates were higher in the treatment arm because participants in this arm were repeatedly asked if they wished to continue, unlike control participants. However, we monitored this monthly, and implemented training to ensure that researchers collecting data were not collecting data differently by trial arm. We also introduced study welcome letters, newsletters and follow‐up reminders for participants, practitioners and researchers in all sites. Evidence of differential dropout appeared to attenuate by the end of the study period after introducing these measures. There were some occasions when a trained IAPT smoking cessation adviser was not available and a participant did not receive their allocated intervention. Another limitation is that IAPT practitioners who provided the acceptability and satisfaction data could have been particularly enthusiastic, compared to those who did not provide data.

We did not reach our initial recruitment target, as recruitment was slower than anticipated. This was because of the COVID‐19 pandemic and because two IAPT services were recommissioned during the study. Recruitment was stopped due to a national injunction to pause non‐COVID‐19 research. On resumption, we shifted to an academic‐led method of approaching potential participants and improved recruitment rates. The recruitment rate varied between Trusts. However, our median recruitment rate per month, by Trust, was two participants, and this rate is higher than the median recruitment rate found in a recent systematic review of 388 trials [36]. We implemented 22 amendments to improve study processes which took, on average, 90 days from submission to local approval. However, we reached 86% of our recruitment target, and our sample size permitted adequate precision to estimate our primary outcome per amendment.

It's possible that socioeconomic factors played a part in participant recruitment and distribution across Trusts. The average IMD score for study participants fell in the third quintile, which is representative of IAPT patients in the highest recruiting Trust. However, in areas with higher deprivation, fewer patients were recruited relative to the case‐load of the respective IAPT services [38, 39].

### Interpretation and implications

The findings of ESCAPE present evidence that integrating smoking cessation treatment into usual IAPT care for common mental illness is acceptable and feasible to IAPT's service users and practitioners. This pilot provides evidence that a full‐sized trial would be possible, and the intervention may be effective. Our smoking cessation intervention was adapted to cover mood management during cessation, given evidence that mood management improves smoking abstinence compared with standard smoking cessation support alone [35].

A Cochrane review compared the effectiveness of combined pharmacotherapy and behavioral treatment against usual care, brief advice, or less intensive behavioral support. At the longest follow‐up, 15.2% (1529 out of 10070) of participants in the intervention group remained abstinent, compared to 8.6% (808 out of 9418) in the control group [40]. In our trial we found that at 6 months, 15.0% (10 of 68) were abstinent in treatment and 6.0% (four of 67) in control. Our quit rates, although derived from a smaller trial, are comparable to other trials that have informed NICE guidelines for smoking cessation [41]. However, the true effect size will not be known until a full‐scale trial is conducted. Given high smoking rates in people with common mental illness, such an intervention integrated into IAPT could substantially reduce smoking prevalence and smoking‐related morbidity and mortality in this population [15].

IAPT services initially approached potential participants but often did not follow through and frequently rotated staff responsible for delivering the intervention (Supporting Information, Appendix). Practitioners explained that the practitioner‐led recruitment model was unsuccessful because they lacked sufficient time to fully explain the study and obtain informed consent. When we compared the practitioner‐led and researcher‐led recruitment models, we found that the researcher‐led approach was more effective. Therefore, in a future trial, we would use a researcher‐led recruitment model to improve participant engagement and ensure a more consistent recruitment process.

The delivery of the intervention in the trial seemed feasible, with a small proportion of participants requesting to discontinue the smoking cessation treatment, and most participants reporting that they were satisfied with the intervention and would recommend it to a friend. There were some potential issues with access to smoking cessation medication, with one‐quarter of participants reporting that medication ‘was not easy to get a hold of’. During the early stages of the trial, prescribers reported concerns that varenicline was contraindicated in people with psychological disorders. Although evidence now suggests that varenicline does not cause psychological disturbance [6, 42], the British National Formulary lists psychological disorders as a caution [43]. Furthermore, in one Trust, general practitioners were not able to prescribe varenicline. IAPT practitioners were happy to deliver the intervention and saw it fitting with their ethos to improve overall wellbeing [28]. More than half of practitioners were satisfied with the delivery of the smoking cessation intervention, and most stated that they would be happy to offer the intervention again, despite evidence that some practitioners in mental health services hold negative attitudes to smoking cessation [44].

Although most practitioners were satisfied with the intervention, there were some intervention barriers. Practitioners reported that additional time or a reduced case‐load would have been beneficial to ensure they could accommodate all their therapy activities, including usual care and the smoking cessation intervention. These qualitative data mapped onto our quantitative data. The initial smoking cessation assessment appointment took longer than expected at an average of 17 minutes; however, follow‐up sessions were shorter at 11 minutes. Another significant barrier was the high turnover of staff in IAPT, which prevented some practitioners from gaining sufficient experience in delivering the intervention. In a future trial, we will address these issues by potentially negotiating with IAPT providers for a reduced case‐load and selecting services with high staff retention. If commissioners decided to offer smoking cessation treatment in IAPT, then staff turnover and case‐load would need to be addressed to allow for successful implementation [45].

In a future trial, considering our 6‐month follow‐up results, a sample size of 598 would be necessary to detect a difference with a P‐value of < 0.05, predicting a 15% quit rate in the treatment group and a 6% quit rate in the control group, with 95% power [37]. If we recruited an average of 33.8 participants per Trust (135/4), we would require 18 (598/33.8) Trusts to participate. Applying our median recruitment rate of two participants per month per Trust, we estimate that each Trust could complete recruitment in 9 months (18/2). However, a significant challenge for a future trial will be the rate at which we recruit Trusts. In this trial we approached Trusts individually, which was time‐consuming and resulted in a recruitment period spanning from June 2018 to August 2021. For a future trial a top‐down recruitment method would be required, with buy‐in from the NHS centrally. This approach would allow all Trusts to commence recruitment simultaneously, rather than sequentially, as happened in this trial.

## CONCLUSION

In this pilot RCT, offering a smoking cessation intervention within IAPT did not cause participants to discontinue their IAPT treatment. Data suggest that the intervention was well received by both participants and IAPT practitioners and the intervention arm achieved higher quit rates, with no evidence of worsening depression or anxiety symptoms compared to control. However, there are some feasibility concerns in running a full‐sized trial in terms of intervention implementation, given practitioner case‐load and retention, and feasibility concerns at the rate of recruiting Trusts. A future trial would need to address these.

## AUTHOR CONTRIBUTIONS

**Gemma M. J. Taylor:** Conceptualisation (lead); data curation (lead); formal analysis (lead); funding acquisition (lead); investigation (lead); methodology (lead); project administration (lead); resources (equal); supervision (lead); writing—original draft (lead). **Katherine Sawyer:** Data curation (supporting); investigation (supporting); project administration (supporting); writing—original draft (supporting). **Pamela Jacobsen:** Investigation (supporting); methodology (supporting); project administration (supporting); writing—original draft (supporting). **Anna Blackwell:** Formal analysis (equal); project administration (supporting); writing—original draft (supporting). **Shadi Daryan:** Formal analysis (supporting); investigation (supporting); project administration (supporting). **Tom P. Freeman:** Investigation (supporting); methodology (supporting). **Chris Metcalfe:** Formal analysis (supporting); investigation (supporting); methodology (supporting); project administration (supporting); supervision (supporting); writing—original draft (supporting). **David Kessler:** Conceptualisation (supporting); funding acquisition (supporting); investigation (supporting); methodology (supporting); supervision (supporting); writing—original draft (supporting). **Marcus R. Munafò:** Conceptualisation (supporting); funding acquisition (supporting); investigation (supporting); methodology (supporting); supervision (supporting); writing—original draft (supporting). **Paul Aveyard:** Conceptualisation (supporting); formal analysis (supporting); funding acquisition (supporting); investigation (supporting); methodology (supporting); supervision (lead); writing—original draft (supporting).

## ETHICS STATEMENT

Ethics approval for this study was received from the NHS Research Ethics Committee on 19/03/2018 (REC reference: 18/SW/0043).

## TRIAL REGISTRATION

ISRCTN99531779 & OSF: https://doi.org/10.17605/OSF.IO/GJ36B

## DECLARATION OF INTERESTS

M. M. and G.T. previously received funding from Pfizer, who manufacture smoking cessation products, for research unrelated to this study. All other authors have nothing to declare. G. T. previously worked at a scientific consultancy providing statistical and research support for pharmaceutical companies to medicines unrelated to this manuscript.

## Supporting information


**Table S1** CONSORT checklist of information to include when reporting a pilot or feasibility trial.
**Table S2** SPIRIT schedule of enrolment, interventions, and assessments.
**Table S3** Table of substantial and non‐substantial amendments submitted for REC and HRA approval.
**Table S4** Participant acceptability and satisfaction with smoking cessation treatment. Data presented are percentages with numerators and denominators for each question and answer on the questionnaire, N.
**Table S5** IAPT practitioner acceptability and satisfaction with smoking cessation treatment. Data presented are percentages with numerators and denominators for each question and answer on the questionnaire.
**Table S6** Categories and subcategories identified using inductive content analysis of the qualitative interviews with IAPT practitioners.
**Table S7** Average smoking cessation intervention duration in minutes. Data presented are mean duration in minutes with standard deviation (SD), and are presented by appointment, N=40.
**Table S8** Smoking cessation medicines/aids recommended to participants by IAPT practitioners at appointment 1. Data presented are frequency of medicine/aid type, with percent (%), N=37.
**Table S9** Smoking cessation medicines/aids recommended to participants by IAPT practitioners at appointment 2. Data presented are frequency of medicine/aid type, as percent (%), N=42.
**Table S10** Blinded researcher response to “At the very start of the telephone call ‐ are you aware of which treatment arm the participant was allocated to?”. Data presented are answer frequency, as a percent (%) with numerator and denominator, at 3‐ and 6‐month follow‐up and by treatment arm.
**Table S11** Blinded researcher response to “At the very start of the telephone call ‐ are you aware of which treatment arm the participant was allocated to?”. Data presented are yes/no answer frequency, as a percent (%) with numerator and denominator, at 3‐ and 6‐month follow‐up and by Trust.
**Table S12** Number of days between randomisation and first IAPT appointment, and first IAPT appointment and 3‐, and 6‐months follow‐up. Data presented are mean differences and standard deviations, with p‐values derived from two sample t‐tests, N=135.
**Table S13** Complete data for main trial outcomes at 3‐month follow‐up from 2020 to 2022. Data presented are percent % complete, with numerators and denominators.
**Table S14** Complete data for main trial outcomes at 6‐month follow‐up from 2020 to 2022. Data presented are percent % complete, with numerators and denominators.
**Table S15** Number of cases where researchers extracted PHQ‐9, GAD‐7, and smoking status data from IAPT clinical contact notes for participants who were lost to follow‐up.
**Table S16** Yearly drop out of trial as per patient request or by difficulty contacting. Data presented are number of participants and percent, by trial arm, N=135.
**Table S17** Overall drop out of trial as per patient request or by difficulty contacting. Data presented are number of participants and percent, by trial arm, N=135.
**Figure S1** IAPT practitioner‐led recruitment model (June 2018 to December 2018).
**Figure S2** Academic researcher‐led recruitment model (January 2018 to August 2021).
**Figure S3** Recruitment counts and cumulative counts for the study recruitment period. Data are presented for each Trust, and across Trusts (N.B. Each Trust had a different recruitment start date due to differences in trial set up dates.

## Data Availability

Data are available via request to the University of Bath's Research Data Archive (https://researchdata.bath.ac.uk/).
