# Supplementary material for: intEgrating Smoking Cessation treatment As part of usual Psychological care for dEpression and anxiety (ESCAPE): A randomised and controlled, multi‐centre, acceptability and feasibility trial with nested qualitative methods
Source: Addiction. 2025 Mar 11;120(5):922–36. doi: 10.1111/add.16718 (PMC11986290; doi:10.1111/add.16718)
Supplement: Supplementary file 1 — Table S1 CONSORT checklist of information to include when reporting a pilot or feasibility trial. Table S2 SPIRIT schedule of enrolment, interventions, and assessments. Table S3 Table of substantial and non‐substantial amendments submitted for REC and HRA approval. Table S4 Participant acceptability and satisfaction with smoking cessation treatment. Data presented are percentages with numerators and denominators for each question and answer on the questionnaire, N. Table S5 IAPT practitioner acceptability and satisfaction with smoking cessation treatment. Data presented are percentages with numerators and denominators for each question and answer on the questionnaire. Table S6 Categories and subcategories identified using inductive content analysis of the qualitative interviews with IAPT practitioners. Table S7 Average smoking cessation intervention duration in minutes. Data presented are mean duration in minutes with standard deviation (SD), and are presented by appointment, N=40. Table S8 Smoking cessation medicines/aids recommended to participants by IAPT practitioners at appointment 1. Data presented are frequency of medicine/aid type, with percent (%), N=37. Table S9 Smoking cessation medicines/aids recommended to participants by IAPT practitioners at appointment 2. Data presented are frequency of medicine/aid type, as percent (%), N=42. Table S10 Blinded researcher response to “At the very start of the telephone call ‐ are you aware of which treatment arm the participant was allocated to?”. Data presented are answer frequency, as a percent (%) with numerator and denominator, at 3‐ and 6‐month follow‐up and by treatment arm. Table S11 Blinded researcher response to “At the very start of the telephone call ‐ are you aware of which treatment arm the participant was allocated to?”. Data presented are yes/no answer frequency, as a percent (%) with numerator and denominator, at 3‐ and 6‐month follow‐up and by Trust. Table S12 Number of days between randomisation and [file ADD-120-922-s001.docx]

**Appendix**

Table of Contents

[1. METHODS 4](#_Toc185857320)

[1.1. CONSORT checklist 5](#_Toc185857321)

[1.2. Recruitment models and model duration employed during ESCAPE. 7](#_Toc185857322)

[1.3. Justification to end recruitment early. 9](#_Toc185857323)

[1.4. Record of trial amendments 10](#_Toc185857324)

[2. RESULTS 13](#_Toc185857325)

[2.1. Barriers and facilitators 13](#_Toc185857326)

[2.1.1. Recruitment into trial 13](#_Toc185857327)

[2.1.2. Implementation of intervention via recordings of intervention sessions 16](#_Toc185857328)

[2.1.3. Differential drop out – researcher retraining. 17](#_Toc185857329)

[2.1.4. Follow-up rates 17](#_Toc185857330)

[2.2. Participant acceptability and satisfaction smoking cessation treatment 18](#_Toc185857331)

[2.3. IAPT practitioner acceptability and satisfaction with the smoking cessation treatment 21](#_Toc185857332)

[2.4. Smoking cessation treatment-related feasibility outcomes 27](#_Toc185857333)

[2.5. Blinded outcome data collection 30](#_Toc185857334)

[2.6. Number of days between randomisation and first IAPT appointment, and first IAPT appointment and 3-, and 6-months follow-up 32](#_Toc185857335)

[2.7. Missing data monitoring for main trial outcomes from 2018 to 2022 33](#_Toc185857336)

[REFERENCES 38](#_Toc185857337)

**List of Tables**

[eTable 1 CONSORT checklist of information to include when reporting a pilot or feasibility trial. 5](#_Toc185857342)

[eTable 2 SPIRIT schedule of enrolment, interventions, and assessments 8](#_Toc185857343)

[eTable 3 Table of substantial and non-substantial amendments submitted for REC and HRA approval. 10](#_Toc185857344)

[eTable 4 Participant acceptability and satisfaction with smoking cessation treatment. Data presented are percentages with numerators and denominators for each question and answer on the questionnaire, N. 18](#_Toc185857345)

[eTable 5 IAPT practitioner acceptability and satisfaction with smoking cessation treatment. Data presented are percentages with numerators and denominators for each question and answer on the questionnaire. 21](#_Toc185857346)

[e Table 6 Categories and subcategories identified using inductive content analysis of the qualitative interviews with IAPT practitioners 25](#_Toc185857347)

[eTable 7 Average smoking cessation intervention duration in minutes. Data presented are mean duration in minutes with standard deviation (SD), and are presented by appointment, N=40. 27](#_Toc185857348)

[eTable 8 Smoking cessation medicines/aids recommended to participants by IAPT practitioners at appointment 1. Data presented are frequency of medicine/aid type, with percent (%), N=37. 28](#_Toc185857349)

[eTable 9 Smoking cessation medicines/aids recommended to participants by IAPT practitioners at appointment 2. Data presented are frequency of medicine/aid type, as percent (%), N=42. 29](#_Toc185857350)

[eTable 10 Blinded researcher response to "At the very start of the telephone call - are you aware of which treatment arm the participant was allocated to?”. Data presented are answer frequency, as a percent (%) with numerator and denominator, at 3- and 6-month follow-up and by treatment arm. 30](#_Toc185857351)

[eTable 11 Blinded researcher response to "At the very start of the telephone call - are you aware of which treatment arm the participant was allocated to?”. Data presented are yes/no answer frequency, as a percent (%) with numerator and denominator, at 3- and 6-month follow-up and by Trust. 31](#_Toc185857352)

[eTable 12 Number of days between randomisation and first IAPT appointment, and first IAPT appointment and 3-, and 6-months follow-up. Data presented are mean differences and standard deviations, with p-values derived from two sample t-tests, N=135. 32](#_Toc185857353)

[eTable 13 Complete data for main trial outcomes at 3-month follow-up from 2020 to 2022. Data presented are percent % complete, with numerators and denominators. 33](#_Toc185857354)

[eTable 14 Complete data for main trial outcomes at 6-month follow-up from 2020 to 2022. Data presented are percent % complete, with numerators and denominators. 34](#_Toc185857355)

[eTable 15 Number of cases where researchers extracted PHQ-9, GAD-7, and smoking status data from IAPT clinical contact notes for participants who were lost to follow-up. 35](#_Toc185857356)

[eTable 16 Yearly drop out of trial as per patient request or by difficulty contacting. Data presented are number of participants and percent, by trial arm, N=135. 36](#_Toc185857357)

[eTable 17 Overall drop out of trial as per patient request or by difficulty contacting. Data presented are number of participants and percent, by trial arm, N=135. 37](#_Toc185857358)

**List of Figures**

[eFigure 1 IAPT practitioner-led recruitment model (June 2018 to December 2018) 7](#_Toc185857359)

[eFigure 2 Academic researcher-led recruitment model (January 2018 to August 2021) 7](#_Toc185857360)

[eFigure 3 Recruitment counts and cumulative counts for the study recruitment period. Data are presented for each Trust, and across Trusts (N.B. Each Trust had a different recruitment start date due to differences in trial set up dates 15](#_Toc185857361)

# METHODS

## CONSORT checklist

eTable 1 CONSORT checklist of information to include when reporting a pilot or feasibility trial.

| **Section/Topic** | **Item No** | **Checklist item** | **Reported on page** |
| --- | --- | --- | --- |
| **Title and abstract** | | | |
|  | 1a | Identification as a pilot or feasibility randomised trial in the title | 1 |
|  | 1b | Structured summary of pilot trial design, methods, results, and conclusions (for specific guidance see CONSORT abstract extension for pilot trials) | 3 |
| **Introduction** | | | |
| Background and objectives | 2a | Scientific background and explanation of rationale for future definitive trial, and reasons for randomised pilot trial | 4 |
|  | 2b | Specific objectives or research questions for pilot trial | 4 |
| **Methods** | | | |
| Trial design | 3a | Description of pilot trial design (such as parallel, factorial) including allocation ratio | 5 |
|  | 3b | Important changes to methods after pilot trial commencement (such as eligibility criteria), with reasons | 8 |
| Participants | 4a | Eligibility criteria for participants | 5 |
|  | 4b | Settings and locations where the data were collected | 5 |
|  | 4c | How participants were identified and consented | 5 |
| Interventions | 5 | The interventions for each group with sufficient details to allow replication, including how and when they were actually administered | 5-6 |
| Outcomes | 6a | Completely defined prespecified assessments or measurements to address each pilot trial objective specified in 2b, including how and when they were assessed | 7-8 |
|  | 6b | Any changes to pilot trial assessments or measurements after the pilot trial commenced, with reasons | 8 |
|  | 6c | If applicable, prespecified criteria used to judge whether, or how, to proceed with future definitive trial | n/a |
| Sample size | 7a | Rationale for numbers in the pilot trial | Published protocol |
|  | 7b | When applicable, explanation of any interim analyses and stopping guidelines | n/a |
| Randomisation: |  |  |  |
| Sequence  generation | 8a | Method used to generate the random allocation sequence | 6 |
|  | 8b | Type of randomisation(s); details of any restriction (such as blocking and block size) | 6 |
| Allocation  concealment  mechanism | 9 | Mechanism used to implement the random allocation sequence (such as sequentially numbered containers), describing any steps taken to conceal the sequence until interventions were assigned | 6 |
| Implementation | 10 | Who generated the random allocation sequence, who enrolled participants, and who assigned participants to interventions | 6 |
| Blinding | 11a | If done, who was blinded after assignment to interventions (for example, participants, care providers, those assessing outcomes) and how | 7 |
|  | 11b | If relevant, description of the similarity of interventions | 5-6 |
| Statistical methods | 12 | Methods used to address each pilot trial objective whether qualitative or quantitative | 8 |
| **Results** | | | |
| Participant flow (a diagram is strongly recommended) | 13a | For each group, the numbers of participants who were approached and/or assessed for eligibility, randomly assigned, received intended treatment, and were assessed for each objective | Figure 1 |
|  | 13b | For each group, losses, and exclusions after randomisation, together with reasons | Figure 1 |
| Recruitment | 14a | Dates defining the periods of recruitment and follow-up | 8 |
|  | 14b | Why the pilot trial ended or was stopped | 8 |
| Baseline data | 15 | A table showing baseline demographic and clinical characteristics for each group | Table 1 |
| Numbers analysed | 16 | For each objective, number of participants (denominator) included in each analysis. If relevant, these numbers  should be by randomised group | Figure 1, Table 4, and appendix |
| Outcomes and estimation | 17 | For each objective, results including expressions of uncertainty (such as 95% confidence interval) for any estimates. If relevant, these results should be by randomised group | Figure 1, Table 4, and appendix |
| Ancillary analyses | 18 | Results of any other analyses performed that could be used to inform the future definitive trial | Appendix |
| Harms | 19 | All important harms or unintended effects in each group (for specific guidance see CONSORT for harms) | 11 |
|  | 19a | If relevant, other important unintended consequences |  |
| **Discussion** | | | |
| Limitations | 20 | Pilot trial limitations, addressing sources of potential bias and remaining uncertainty about feasibility | 12 |
| Generalisability | 21 | Generalisability (applicability) of pilot trial methods and findings to future definitive trial and other studies | 11-12 |
| Interpretation | 22 | Interpretation consistent with pilot trial objectives and findings, balancing potential benefits and harms, and  considering other relevant evidence | 11-14 |
|  | 22a | Implications for progression from pilot to future definitive trial, including any proposed amendments | 12-14 |
| **Other information** | | |  |
| Registration | 23 | Registration number for pilot trial and name of trial registry | 5 |
| Protocol | 24 | Where the pilot trial protocol can be accessed, if available | 5 |
| Funding | 25 | Sources of funding and other support (such as supply of drugs), role of funders | 2 |
|  | 26 | Ethical approval or approval by research review committee, confirmed with reference number | 2 |

## Recruitment models and model duration employed during ESCAPE.

eFigure 1 IAPT practitioner-led recruitment model (June 2018 to December 2018)

eFigure 2 Academic researcher-led recruitment model (January 2018 to August 2021)

eTable 2 SPIRIT schedule of enrolment, interventions, and assessments

|  | Pre-randomisation | Randomisation | Pre-treatment | Post-Enrolment | | | | Close |
| --- | --- | --- | --- | --- | --- | --- | --- | --- |
| Time point | t-1 | t0 | t1 | t2 | t3-11 | t12 | t13 | t14 |
| Appointment details |  |  |  | IAPT appt. 1 | IAPT appt. 2-10 | 3-months | 6-months |  |
| Enrolment: |  |  |  |  |  |  |  |  |
| Eligibility screen | ✓ |  |  |  |  |  |  |  |
| Informed consent | ✓ |  |  |  |  |  |  |  |
| Allocation | ✓ |  |  |  |  |  |  |  |
| Interventions: |  |  |  |  |  |  |  |  |
| Intervention |  |  |  |  |  |  |  |  |
| Control |  |  |  |  |  |  |  |  |
| Assessments: |  |  |  |  |  |  |  |  |
| Age | ✓ |  |  |  |  |  |  |  |
| Sex | ✓ |  |  |  |  |  |  |  |
| Education | ✓ |  |  |  |  |  |  |  |
| Ethnicity | ✓ |  |  |  |  |  |  |  |
| PHQ-9 |  |  | ✓ | ✓ | ✓ | ✓ | ✓ |  |
| GAD-7 |  |  | ✓ | ✓ | ✓ | ✓ | ✓ |  |
| Heaviness of Smoking Index |  |  | ✓ | ✓ |  | ✓ | ✓ |  |
| Cigarettes per day |  |  | ✓ | ✓ |  | ✓ | ✓ |  |
| Previous quit attempts |  |  | ✓ |  |  |  |  |  |
| Mental health co-morbidities |  |  |  | ✓ | ✓ |  |  |  |
| Planned, completed, and missed IAPT appointments |  |  |  | ✓ | ✓ | ✓ | ✓ |  |
| IAPT treatment status (active/discontinued/completed/discharged) |  |  |  | ✓ | ✓ | ✓ | ✓ |  |
| Smoking cessation treatment session (completed/not completed, and duration) |  |  |  | ✓ | ✓ |  |  |  |
| Retention in smoking cessation treatment (continued/discontinued) |  |  |  | ✓ | ✓ | ✓ | ✓ |  |
| Smoking cessation medication usage (type of medicine/e-cig use) |  |  |  | ✓ | ✓ | ✓ | ✓ |  |
| Self-reported 7-day point-prevalence smoking cessation |  |  |  |  | ✓ | ✓ | ✓ |  |
| Biologically validated 7-day point-prevalence smoking cessation |  |  |  |  |  | ✓ | ✓ |  |
| Stop Smoking Service Client Satisfaction Survey |  |  |  |  |  | ✓ | ✓ |  |
| Clinician Self-Report Intervention Acceptability and Satisfaction Questionnaires |  |  |  |  |  |  |  | ✓ |
| Adverse events |  |  | ✓ | ✓ | ✓ | ✓ | ✓ | ✓ |

## Justification to end recruitment early.

We wrote our justification on August 1, 2021, and decided to stop recruitment August 31, 2021.

Our previous sample size calculation (157 participants - recruitment target) gave us precision to estimate that 40% or more participants will continue with smoking cessation intervention in the treatment arm after 36%^1^ attrition from baseline, giving us a 95% confidence interval of 26% to 55%. (Stata command - cii proportions 50 20)

*Complete case precision estimate*

At the time of deciding to end recruitment we had enrolled 128 participants in the trial, assuming 36% attrition^1^ (46/128=36%), therefore leaving us with 82 (128-46) participants at follow-up (82 total, 41 per arm). If 40% (16/41) of remaining participants continue with smoking cessation treatment in the treatment arm, this gives us a 95% CI of 24% to 55%. (Stata command - cii proportions 41 16).

*Intention to treat precision estimate.*

We currently have 128 enrolled in the trial. If 25% (16/64) of remaining participants continue with smoking cessation treatment in the treatment arm, this gives us a 95% CI of 24% to 55%. (Stata command - cii proportions 64 16).

## Record of trial amendments

eTable 3 Table of substantial and non-substantial amendments submitted for REC and HRA approval.

| **Amendment type (non-substantial (NSA) or substantial (SA) and number)** | **Description** | **Date REC approval applied for/Or letter of valid application received** | **Final date that local trust approval received (take latest date of all trusts)** | **N days between date applied and approval received** |
| --- | --- | --- | --- | --- |
| SA1 | IAPT sites have requested that study posters | 04-Jan-18 | 30-Jul-18 | 207 |
| SA2 | Oxford site only. Change in NRT delivery. | 16-Oct-18 | 23-Jan-19 | 99 |
| NSA2 | Recruitment date extended to September 31, 2019. | 17-Oct-18 | 17-Nov-18 | 31 |
| NSA3 | Vouchers for recruiting assessors | 29-Nov-18 | 30-Apr-19 | 152 |
| SA3 | Change in Sponsorship | 18-Jan-19 | 21-May-19 | 123 |
| NSA5 | Participant information sheets will be posted to potentially eligible participants who have joined the service | 13-Mar-19 | 09-May-19 | 57 |
| SA6 | Adding staff survey to explore implementation barriers/opportunities in primary and secondary care | 05-Apr-19 | 28-Jun-19 | 84 |
| NSA6 | Extending recruitment date until Aug 31 2020 | 18-Apr-19 | 22-Aug-19 | 126 |
| SA7 | •Additional follow-up data collected (this is reflected in the consent form, client information sheets, CFR forms and protocol version 9.0): Adding a 6-month follow-up to meet smoking cessation gold standard for RCTs. See West, R., et al., Outcome criteria in smoking cessation trials: proposal for the common standard. Addiction, 2005. 100, 299-303. Adding the GAD-7 at 3 and 6-months follow-up. Questions added to follow-up and RECAP: Has the participant stated they wish to stop participating in the study? Has the follow-up not been completed due to difficulty contacting the client? Have you accessed a stop smoking service as either part of the intervention arm alongside IAPT treatment or outside of these sessions? •Adding an additional recruitment stage. The IAPT practitioner-led recruitment model was failing to recruit as PWPs were finding it too burdensome. Revised the recruitment process with the Trust RD team, who think that the responsibility will be better placed with the trust RD team. The new recruitment process will be as follows: i) PWPs will ask clients during telephone-based assessments whether they smoke and if they are interested in receiving a telephone call about the study from a trust researcher ii) if the client is interested, the PWP will send the client's IAPTUS/PCMIS ID to trust RA/CSOs via NHS email to discuss the study further. (Reflected in protocol version 9.0) •To improve follow-up rates, participants will be offered a £10 voucher via email or recorded delivery. (Reflected in consent form, client information sheets, CRF forms and protocol version 9.0). •Adding "Participant information pack", as previously approved in original protocol but was not attached to IRAS. | 31-May-19 | 22-Aug-19 | 83 |
| NSA8 | Adding new NHS sites for recruitment and intervention delivery | 20-Jun-19 | 22-Aug-19 | 63 |
| NSA9 | Adding North Bristol Trust as a site | 01-Aug-19 | 27-Sep-19 | 57 |
| NSA10 | •Rewording NRT procedure (there was confusion with Oxford site) •Rewording settings to allow for additional sites •Removed addresses from documents to permit use by new sites •New sites for ESCAPE trial: BANES (Avon and Wiltshire Partnership Trust), and Dudley and Walsall Mental Health Partnership Trust •New sites for Staff Survey: Gloucestershire Hospitals NHS Foundation Trust, North Bristol Trust, Primary Care West England, Research 2gether, Royal United Hospital Bath NHS Trust | 12-Sep-19 | 29-Jan-20 | 139 |
| NSA11 | •Online staff survey 1st October, 2gether and Gloucestershire Care Services merged to become Gloucestershire Health and Care NHS Foundation Trust •New site for ESCAPE trial: Northeast London NHS Foundation Trust •Added PIs for NELFT and Dudley and Walsall •Added Co-PI for AWP •Reworking NRT procedure (there was confusion with Oxford site) •Adding BANES NRT and Varenicline forms | 20-Nov-19 | 29-Jan-20 | 70 |
| NSA12 | •Clarifying intervention recording procedures and data storage •Updated lead PWP/Researcher contact details as per staff changes in trusts •Clarifying data collection and data storage procedures | 18-Mar-20 | 21-May-20 | 64 |
| SA13 | •Follow-up reminder letters - to improve follow-up rates, researchers can contact participants outside of normal working hours if not contactable during working hours. If a participant is difficult to follow-up, the CI must be informed.  •Researchers should send participants a "thank you for participating postcard" (in sealed envelope so as not to disclose participation) 1-month after baseline. •Researchers should also send participants a follow-up reminder letter during the trial period to remind participants they will be contacted for follow-up •Consent and PIS for questionnaire for PWPs/practitioners: adding a consent form for questionnaire for PWPs/practitioners to complete when they've finished delivering the intervention. The questionnaire has already been approved but forgot to add a consent form for this. Pre-existing PIS has been altered for follow-up interviews with PWPs/practitioners. | 30-Jan-20 | 21-May-20 | 112 |
| NSA14 | •Principal Investigator for AWP changed to <name> from <name> and <name> as they no longer work there.  •Clarifying follow-up and missing data procedures. •ESCAPE online survey - adding 67 sites that sent EOI | 25-Mar-20 | 19-May-20 | 55 |
| SA15 | •Due to COVID - can no longer collect expired carbon-monoxide levels. To collect saliva samples and analyse cotinine levels (metabolite of nicotine) to verify cessation.  •Due to COVID recruitment is on hold in two sites and would therefore like to extend recruitment to August 31, 2021.  •Dudley and Walsall NHS FT is now "Black Country Healthcare NHS Foundation Trust" | 01-May-20 | 03-Jul-20 | 63 |
| SA16 | •Adding an ESCAPE newsletter for participants, researchers, and IAPT practitioners to optimise study engagement.  •ESCAPE sub study - online: adding Eastern Primary Care CRN as a site. | 19-Aug-20 | 18-Nov-20 | 91 |
| SA17 | Adding "late voucher letter" | 15-Sep-20 | 18-Nov-20 | 64 |
| SA18 | Dr Tom Freeman is Chief investigator cover for Dr Gemma Taylor's maternity leave | 04-Dec-20 | 19-Feb-21 | 77 |
| NSA19 | Minor change to "CFR 8_short version for PWP treatment appt 1-10". Taking off question that's irrelevant to ask during consultations. Researchers will receive this information at 3- and 6-month follow-up. | 08-Dec-20 | 20-Apr-21 | 133 |
| NSA20 | Trust name change from Black Country Healthcare NHS Foundation Trust to Dudley Integrated Health and Care NHS Trust | 16-Apr-21 | 09-Jun-21 | 54 |
| NSA21 | Extending trial close date to Dec 31, 2022. Some patients who were enrolled in August are still on IAPT waiting list for their first appointment. | 25-Jan-22 | 06-Apr-22 | 90 |

# RESULTS

## Barriers and facilitators

### Recruitment into trial

In the trial there were differences in recruitment rates across Trusts, Oxford Health NHS Trust (OX) had the highest recruitment rate and total compared to the other three Trusts, Northeast London Foundation Trust (NELFT), Avon and Wiltshire Mental Health Partnership Trust (AWP) and Dudley Integrated Health and Care Trust (DIHCT), several potential explanations for why OX experienced a higher recruitment rate are discussed. In OX the research processes were well integrated with the IAPT services. Contact with the researcher was classed as engagement, so that non-contact with the researcher was classed as disengagement with the IAPT service. For example, if a client expressed interest in research at their review appoint in IAPT treatment pathway, then the researcher contacts them as a potential recruit. If the client does not answer/engage with the researcher, then the client is sent a “disengagement letter” from the IAPT service and followed up by the IAPT practitioner, prompting them to respond to the research team to then progress into treatment. The disengagement letter is framed in the way of finding out if the client would or would not like to take part in the trial so that they can be allocated to the appropriate practitioner and did not prevent access to treatment. This method could have increased recruitment as it prevented potentially eligible participants, who had agreed to hear about the study, starting treatment without having responded to the research team. This method was not implemented in other Trusts (NELFT, AWP, DIHCT), as it did not fit with their IAPT service organisational culture or processes.

Another method which could have promoted recruitment in OX is the timing of sending out the study PIS. After initial referral stages, (Where IAPT practitioners asked the client "do you smoke" "would like a research call or to hear about research") the OX researchers would make their first contact with the client to discuss the ESCAPE study, if participants did not answer the phone, the researcher would leave a voicemail/email explaining the study and would also send the PIS. Potentially eligible participants would receive the PIS whether the participant answered the research team phone call or not, this was deemed appropriate as the person will have previously agreed to hear about research. This could have encouraged recruitment as participants were aware of the study, and the researcher’s name and number, on the second contact attempt, and so could have been more likely to answer the researcher recruitment call and thus be recruited. In other Trusts (NELFT, AWP, DIHCT) researchers would continue to try and phone the potentially eligible participants and would only send the PIS once they had gotten in contact with the participant, thus there were many people who researchers could not get in contact with and who didn’t receive the study PIS. This method was not implemented in other Trusts (NELFT, AWP, DIHCT), as it did not fit with their research team organisational culture or processes.

Some other Trusts experienced barriers to recruitment, for example in AWP, the Bristol IAPT service were recommissioned in May 2019 to Vita Health. In this recommission 3 participants were randomised but then did not receive baseline assessments or the allocated treatment, due to issues with data transfer, we were however able to follow-up 2 of these participants. Bristol closed as a site in 2019 and did not reopen due to the recommissioning.

After the recommission B&NES Primary Care IAPT Service in December 2019 was opened as another service within AWP, however no participants were recruited, and it was realised that this was due to issues with the timing of recruitment in the treatment pathway, where potentially eligible participants had already started therapy before they could be recruited by the research team. The recruitment process was then adapted with potential participants being identified and contacted earlier on in the treatment pathway, once adapted, recruitment was reopened for a second phase.

At the time of the second recruitment phase the B&NES Primary Care IAPT Service had many trainees IAPT practitioners, trainees were ineligible to be trained in smoking cessation for the trial. Trainees had priority over ‘simple’ cases, some of which were potentially eligible participants, thus participants that could have been recruited were allocated to trainees, before being enrolled into the study. Moreover, Bath was also experiencing unusually low referral rates from January 2021 onwards, and it is thought this could have been due to the COVID-19 pandemic, where less people were accessing GP’s and being referred. It is unclear if this same problem was seen in other Trusts, or why it was occurring in Bath only.

DIHCT was another Trust that experienced recruitment barriers, DIHCT was experiencing a steady recruitment rate (1-2 per month), until January 2021 when the Trust had to temporarily close due to queries with the service level agreement when the trusts Black Country Healthcare NHS Foundation Trust and Dudley and Walsall Mental Health Partnership NHS Trust Merged to form DIHCT. Recruitment into the study was closed from January 2021 until end of April 2021. As well as this, as of August 2021, Dudley no longer offered IAPT as a service.

eFigure 3 Recruitment counts and cumulative counts for the study recruitment period. Data are presented for each Trust, and across Trusts (N.B. Each Trust had a different recruitment start date due to differences in trial set up dates

### Implementation of intervention via recordings of intervention sessions

There were various issues with obtaining recording of interventions to assess fidelity and intervention implementation, with different issues in different Trusts. It is important to note, that most participants 4/135 consented to and were onboard with having their sessions recorded, the barriers were encountered at the service level, not the participant level. Across the Trusts, one common barrier was that in January 2021, the NHS governance team no longer permitted the use of Dictaphones for recording treatment sessions in IAPT services, this made it difficult to have the equipment to record the sessions in almost all Trusts and caused delays.

NELFT: NELFT had the appropriate approvals from information governance to record intervention sessions. NELFT however had difficulty obtaining equipment for recording after the NHS prohibited Dictaphones, this was exacerbated as many IAPT practitioners were working from home due to the COVID-19 Pandemic. However, NELFT were able to obtain a verbal (in April 2021) written (exception permitting the use of Dictaphones for their use of recording intervention sessions in the ESCAPE trial, from their information governance team. After some investigation, NELFT also had the means of securely transferring audio files from the trust research team to the University of Bath research team using their Media Hub. In May 2021 options for Dictaphones were sent to NELFT information governance team for review, the selected devices did not have the suitable encryption, the IG team suggested a Dictaphone, but this was beyond the trial budget. In June 2021 some Dictaphones in NELFT were identified that meet the criteria for encryption, however a software was needed on the device to set a pin to make them secure. In August 2021 it was confirmed that, despite IT trying various things and seeking advice from the manufacturer, the team were unable to download the software on the Dictaphone and get them to work and so were unable to use them, and unable to record intervention sessions.

DIHCT: Dudley had the appropriate approvals from information governance to record intervention sessions and the equipment to record sessions. The challenge in Dudley was finding an approved method to securely transfer files from Dudley trust to the University of Bath research team. Another challenge was that by the time appropriate approvals to record sessions were granted, the Trust was closed for recruitment and there were no participants in treatment, thus no sessions to record (as of July 2021).

AWP: Recording intervention sessions in AWP was also a challenge. The Bath IAPT service had the equipment to record the sessions and procedure to transfer audio files to the University of Bath Research team. Despite the Bath IAPT team being onboard with the recordings, the AWP information governance team denied 3 requests over a 1.5-year period to record the sessions and set up the file transfer pathway with the final denial in March 2021. The only available solution to assess fidelity was for a University of Bath researcher to observe intervention sessions remotely and assess fidelity and intervention implementation, treatment sessions were not randomly selected to limited availability of data.

OX: In OX, edits to the PIs and consent form were requested to provide more clarity on recording intervention sessions, NSA12 was made and completed in January 2020. Covid-19 then hit and any addition activities, such as recording sessions was paused. When raised again, the information governance team and service lead denied requests to record the IAPT intervention sessions. There was also no equipment available to record sessions because IAPT practitioners were working from home in the COVID-19 Pandemic, this was confirmed in December 2020. OX did however have a potential file transfer method. The only available solution to assess fidelity was for a University of Bath researcher to observe intervention sessions remotely and assess fidelity and intervention implementation, treatment sessions were not randomly selected to limited availability of data. Only a couple of sessions were observed due to issues with consent processes which caused delays to the approval of observations which in turn left only few participants still in treatment and available to observe.

| **Trust** | **Approvals** | **Means to record** | **Transfer method** |
| --- | --- | --- | --- |
| Northeast London Foundation Trust (NELFT) | Yes | No | Yes |
| Dudley Integrated Health and Care Trust Integrated Health and Care Trust (DIHCT) | Yes | Yes | Challenging |
| Avon and Wiltshire Mental Health Partnership Trust (AWP) | No | Yes | Yes |
| Oxford Health NHS Trust (OX) | No | No | Potentially |

### Differential drop out – researcher retraining.

In the trial, there were high levels of participants withdrawing in the treatment arm, it was soon realised that this was because only intervention participants were being asked if they were still ok to carry on in the trial at each IAPT appointment, whereas control participants were receiving usual care and not asked this question at each IAPT appointment. In December 2020 (approved in April 2021 due to miscommunication), the protocol was therefore amended and the question checking if participants were still ok to carry on was removed from IAPT treatment CRF’s. This differential asking is likely to have led to differential drop out and is more evident in Trusts who had been recruiting for longer before the amendment was actioned, for example OX vs NELFT.

### Follow-up rates

In December 2019 it was noted that follow-up rates were low in OX and AWP (NELFT and DIHCT were not following up at this time), at worst a 46% and at best a 62% retention rate. Compared to one trial in IAPT (73 % follow-up rate^2^) and smoking cessation trials in people with depression (64% retention rate^3^) this follow-up rate is low. To improve follow-up rates, we:

- Offered top-up training for data collection & follow-ups.
- Started sending missing data reports to Trusts – updated biweekly highlighting missing data, and follow-ups due.
- Imputed missing data from PCMIS/IAPTUS within 1-month either side of follow-up. i.e., PHQ, GAD, and smoking status (if IAPT practitioner remembers to record this and data are available)
- Added out of hours follow-ups.
- Added study “welcome” letters.
- Added “follow-up reminder” letters.
- Added prompts on REDCAP for CSOs/RAs at baseline and follow-ups to remind participants about follow-ups.
- Added a study newsletter.
- Ensured that we correctly classed people as “discontinued”.

Follow-up rates improved to September 2020, but since then, 3-months follow-up rates have improved, but we were unable to recuperate from the original 6-month loss-to-follow-up (Section 2.7). The increase in missing data was discussed at a trial management meeting in September 2021 and a new action plan to improve missing data was implemented in which academic researchers would be reintroduced to support follow-ups, at this point we were able to achieve improved follow-up response rates.

## Participant acceptability and satisfaction smoking cessation treatment

eTable 4 Participant acceptability and satisfaction with smoking cessation treatment. Data presented are percentages with numerators and denominators for each question and answer on the questionnaire, N.

| **Answers** | **Question** | **%** |
| --- | --- | --- |
|  | **Since you started therapy with IAPT have you received smoking cessation support?** |  |
| No | 18 | 43.9 |
| Yes | 23 | 56.1 |
| **Total** | 41 |  |
|  |  |  |
|  | **How long did you have to wait before your first appointment?** |  |
| Less than 30 days | 14 | 66.7 |
| Between 30 and 60 days | 4 | 19.0 |
| Between 91 and 120 days | 2 | 9.5 |
| More than 120 days | 1 | 4.8 |
| **Total** | 21 |  |
|  |  |  |
|  | **Overall, how satisfied are you with the support you have received to stop smoking?** |  |
| Unsatisfied | 1 | 4.8 |
| Unsure | 1 | 4.8 |
| Satisfied | 7 | 33.3 |
| Very Satisfied | 12 | 57.1 |
| **Total** | 21 |  |
|  |  |  |
|  | **Would you recommend this service to other smokers who want to stop smoking?** |  |
| Unsure | 5 | 23.8 |
| Yes | 16 | 76.2 |
| **Total** | 21 |  |
|  |  |  |
|  | **In the event that you started smoking again would you go back to the service for help?** |  |
| No | 2 | 9.5 |
| Unsure | 4 | 19.0 |
| Yes | 15 | 71.4 |
| **Total** | 21 |  |
|  |  |  |
|  | **If you returned to the service for help with stopping smoking in the future, do you think you’d be welcome?** |  |
| Unsure | 2 | 9.5 |
| Yes | 19 | 90.5 |
| **Total** | 21 |  |
|  |  |  |
|  | **Have you smoked since your last appointment with the service?** |  |
| No, not a single puff | 9 | 42.9 |
| Yes, just a few puffs | 1 | 4.8 |
| Yes, 1-5 cigarettes | 3 | 14.3 |
| More than 5 cigarettes | 8 | 38.1 |
| **Total** | 21 |  |
|  |  |  |
|  | **When you contacted the service were you given an appointment date or told how long you would have to wait?** |  |
| No | 3 | 14.3 |
| Unsure | 1 | 4.8 |
| Yes | 17 | 81.0 |
| **Total** | 21 |  |
|  |  |  |
|  | **Was the length of time you had to wait for your first appointment acceptable to you?** |  |
| No | 5 | 25.0 |
| Yes | 15 | 75.0 |
| **Total** | 20 |  |
|  |  |  |
|  | **Was there contact from the stop smoking service before your appointment to encourage you to attend?** |  |
| No | 7 | 33.3 |
| Unsure | 2 | 9.5 |
| Yes | 12 | 57.1 |
| **Total** | 21 |  |
|  |  |  |
|  | **Are the appointment times you were given convenient for you?** |  |
| Unsure | 2 | 9.1 |
| Yes | 20 | 90.9 |
| **Total** | 22 |  |
|  |  |  |
|  | **How satisfied are you with how supportive staff have been?** |  |
| Very Unsatisfied | 1 | 4.5 |
| Unsatisfied | 1 | 4.5 |
| Satisfied | 4 | 18.2 |
| Very Satisfied | 16 | 72.7 |
| **Total** | 22 |  |
|  |  |  |
|  | **How helpful has the information and advice that staff have given to you during your appointment?** |  |
| Unsure | 1 | 4.5 |
| Helpful | 7 | 31.8 |
| Very helpful | 14 | 63.6 |
| **Total** | 22 |  |
|  |  |  |
|  | **How helpful has the written information that staff have given to you been?** |  |
| Very Unhelpful | 1 | 4.8 |
| Unhelpful | 1 | 4.8 |
| Unsure | 5 | 23.8 |
| Helpful | 5 | 23.8 |
| Very helpful | 9 | 42.9 |
| **Total** | 21 |  |
|  |  |  |
|  | **Was the information that you were given about the choice of medication helpful?** |  |
| Unsure | 1 | 4.8 |
| Yes | 19 | 90.5 |
| Did not use medication | 1 | 4.8 |
| **Total** | 21 |  |
|  |  |  |
|  | **Was it easy to get hold of your smoking cessation medicine once you had chosen which medication you wanted?** |  |
| No | 5 | 23.8 |
| Unsure | 1 | 4.8 |
| Yes | 12 | 57.1 |
| Did not use medication | 3 | 14.3 |
| **Total** | 21 |  |

## IAPT practitioner acceptability and satisfaction with the smoking cessation treatment

eTable 5 IAPT practitioner acceptability and satisfaction with smoking cessation treatment. Data presented are percentages with numerators and denominators for each question and answer on the questionnaire.

| **Answers** | **Questions** | **%** |
| --- | --- | --- |
|  | **Satisfaction- I was satisfied with the delivery of the smoking cessation intervention** |  |
| Disagree | 3 | 16.7 |
| Neither agree or disagree | 4 | 22.2 |
| Agree | 11 | 61.1 |
| **Total** | 18 | 100.0 |
|  |  |  |
|  | **Satisfaction- The length of sessions was adequate** |  |
| Strongly disagree | 4 | 22.2 |
| Disagree | 7 | 38.9 |
| Neither agree or disagree | 4 | 22.2 |
| Agree | 3 | 16.7 |
| **Total** | 18 | 100.0 |
|  |  |  |
|  | **Satisfaction- The structure of sessions was logical** |  |
| Disagree | 1 | 5.6 |
| Neither agree or disagree | 4 | 22.2 |
| Agree | 8 | 44.4 |
| Strongly agree | 5 | 27.8 |
| **Total** | 18 | 100.0 |
|  |  |  |
|  | **Satisfaction- The intervention manual was easy to use** |  |
| Disagree | 3 | 16.7 |
| Neither agree or disagree | 3 | 16.7 |
| Agree | 8 | 44.4 |
| Strongly agree | 4 | 22.2 |
| **Total** | 18 | 100.0 |
|  |  |  |
|  | **Satisfaction- I kept to the intervention framework in the sessions** |  |
| Disagree | 2 | 11.1 |
| Neither agree or disagree | 4 | 22.2 |
| Agree | 11 | 61.1 |
| Strongly agree | 1 | 5.6 |
| **Total** | 18 | 100.0 |
|  |  |  |
|  | **Satisfaction- The intervention seemed to meet my patient’s needs** |  |
| Disagree | 2 | 11.1 |
| Neither agree or disagree | 5 | 27.8 |
| Agree | 7 | 38.9 |
| Strongly agree | 4 | 22.2 |
| **Total** | 18 | 100.0 |
|  |  |  |
|  | **Satisfaction- I would be happy to offer this intervention again** |  |
| Disagree | 2 | 11.1 |
| Neither agree or disagree | 1 | 5.6 |
| Agree | 9 | 50.0 |
| Strongly agree | 6 | 33.3 |
| **Total** | 18 | 100.0 |
|  |  |  |
|  | **AIM- The smoking cessation intervention meets my approval** |  |
| Disagree | 2 | 14.3 |
| Neither agree nor disagree | 3 | 21.4 |
| Agree | 7 | 50.0 |
| Completely agree | 2 | 14.3 |
| **Total** | 14 | 100.0 |
|  |  |  |
|  | **AIM- smoking cessation intervention is appealing to me** |  |
| Completely disagree | 1 | 7.1 |
| Disagree | 2 | 14.3 |
| Neither agree nor disagree | 1 | 7.1 |
| Agree | 7 | 50.0 |
| Completely agree | 3 | 21.4 |
| **Total** | 14 | 100.0 |
|  |  |  |
|  | **AIM- I like the smoking cessation intervention** |  |
| Disagree | 3 | 21.4 |
| Neither agree nor disagree | 3 | 21.4 |
| Agree | 4 | 28.6 |
| Completely agree | 4 | 28.6 |
| **Total** | 14 | 100.0 |
|  |  |  |
|  | **AIM- I welcome the smoking cessation intervention** |  |
| Completely disagree | 1 | 7.1 |
| Disagree | 3 | 21.4 |
| Neither agree nor disagree | 2 | 14.3 |
| Agree | 3 | 21.4 |
| Completely agree | 5 | 35.7 |
| **Total** | 14 | 100.0 |
|  |  |  |
|  | **IAM- The smoking cessation intervention seems fitting** |  |
| Completely disagree | 1 | 7.1 |
| Disagree | 1 | 7.1 |
| Neither agree nor disagree | 3 | 21.4 |
| Agree | 7 | 50.0 |
| Completely agree | 2 | 14.3 |
| **Total** | 14 | 100.0 |
|  |  |  |
|  | **IAM- The smoking cessation intervention seems suitable** |  |
| Completely disagree | 1 | 7.1 |
| Disagree | 1 | 7.1 |
| Neither agree nor disagree | 1 | 7.1 |
| Agree | 8 | 57.1 |
| Completely agree | 3 | 21.4 |
| **Total** | 14 | 100.0 |
|  |  |  |
|  | **IAM- The smoking cessation intervention seems applicable** |  |
| Completely disagree | 1 | 7.1 |
| Disagree | 1 | 7.1 |
| Neither agree nor disagree | 3 | 21.4 |
| Agree | 7 | 50.0 |
| Completely agree | 2 | 14.3 |
| **Total** | 14 | 100.0 |
|  |  |  |
|  | **IAM- The smoking cessation intervention seems like a good match** |  |
| Disagree | 3 | 21.4 |
| Neither agree nor disagree | 3 | 21.4 |
| Agree | 4 | 28.6 |
| Completely agree | 4 | 28.6 |
| **Total** | 14 | 100.0 |
|  |  |  |
|  | **FIM- The smoking cessation intervention seems implementable** |  |
| Completely disagree | 2 | 14.3 |
| Disagree | 3 | 21.4 |
| Neither agree nor disagree | 2 | 14.3 |
| Agree | 6 | 42.9 |
| Completely agree | 1 | 7.1 |
| **Total** | 14 | 100.0 |
|  |  |  |
|  | **FIM- The smoking cessation intervention seems possible** |  |
| Disagree | 4 | 28.6 |
| Neither agree nor disagree | 2 | 14.3 |
| Agree | 7 | 50.0 |
| Completely agree | 1 | 7.1 |
| **Total** | 14 | 100.0 |
|  |  |  |
|  | **FIM- The smoking cessation intervention seems doable** |  |
| Completely disagree | 1 | 7.1 |
| Disagree | 5 | 35.7 |
| Neither agree nor disagree | 3 | 21.4 |
| Agree | 3 | 21.4 |
| Completely agree | 2 | 14.3 |
| **Total** | 14 | 100.0 |
|  |  |  |
|  | **FIM- The smoking cessation intervention seems easy to use** |  |
| Disagree | 5 | 35.7 |
| Neither agree nor disagree | 2 | 14.3 |
| Agree | 6 | 42.9 |
| Completely agree | 1 | 7.1 |
| **Total** | 14 | 100.0 |

e Table 6 Categories and subcategories identified using inductive content analysis of the qualitative interviews with IAPT practitioners

| ***Category*** | ***Subcategory*** | ***Example quotes*** |
| --- | --- | --- |
| **Smoking cessation as an interesting professional opportunity** | | "I've always been very interested in in addiction and the treatment of addiction with CBT …it was a great opportunity to actually yeah apply a treatment to help somebody struggling with an addiction within the service." London service, female PWP (5 years’ experience), age 35 |
| **Facilitators of intervention delivery** | Smoking as part of the bigger picture | "I think CBT’s really good in the sense that it’s very structured anyway so all appointments will have a standard kind of agenda that you're using, so essentially I’d agenda both, you have a smoking check-in, thinking about how that’s going and then separate the latter half of the session into any other work that you're doing. I guess as it’s behaviour activation, those things complement so well that they can kind of be thought about together whereas if you're working with an anxiety, it’s very different so you would do smoking check-in and then behavioural change but behavioural change with an anxiety disorder is very different, so you would come onto do some graded exposure on a phobia, it’s kind of got nothing to do with the smoking side of things, that’s very separate whereas if you're working with depression, they can be quite similar. I did find with quite a few of my patients, the majority of the session was talked about the smoking side of things because their mood was improving so much through that, it was almost like you didn’t have to do that much extra work alongside it." Southwest England service, female PWP (4 years’ experience), age 29 |
|  | Confidence | "Initially I felt like it was quite a lot to learn actually, but all of the materials I was sent for the training and being part of the trial was really helpful, it was very detailed. So, at the beginning, I’m the kind of person that I need to feel confident in it before I do it, so I did spend quite a lot of time just reading over the materials and I made a few notes on my own Word document so I had it there with me at the start so that when I was explaining it to them I had it on the screen in case I lost my way. But after I’d done it a few times it was quite second nature and then I was just able to confidently discuss it. But initially I did feel that I needed to do quite a lot of reading and prep just so I felt confident really. But all the materials where there provided, so it was really helpful to have all of that. Southwest England service, female PWP (3.5 years’ experience), age 31 |
| **Client readiness to make a change** | Understanding and accepting the link between smoking and mental health | "I felt like it came a bit like something new ... a lot [of] people still think that you know you. If you're stressed, you use whether to smoke like so I think there's a lot of lack of knowledge about it, and that's when the you know the this research is very helpful because people really needs to hear that, um, smoking is not gonna make you less stressed or is isn’t gonna help with your low mood, but people still believe that so that’s, yeah, it's quite a contrast what they think versus what you tell them, so that also takes time. I think for them to accept it so... we did get somewhere within the session, um, and by talking about it, they seem to get the rationale. But then obviously I think this myth is really strong in in this society and um you cannot really counteract that with one session" London service, female PWP (5 years’ experience), age 35 |
|  | Motivation and commitment | "And also just reiterating, towards the beginning we would always talk through their reasons why they want to stop, helping them to remember about that as well. Because I think sometimes people might have more short-term stresses or situations where they just think, ‘This is too important, I just can’t not smoke at the moment’, whereas actually reminding them of the longer-term benefits of why they’re doing and why they want to make those changes was quite helpful as well." Southwest England service, female PWP (3.5 years’ experience), age 31 |
|  | Reciprocal impact of smoking cessation and mental health treatment | "Then actually with most of my patients as well, they often recovered on the standard minimum dataset for anxiety and depression once they’d given up smoking, which I thought was really interesting. I think one of the reasons for that is because as they gave up, it almost like gave them a confidence boost which then kick started their motivation and some of the behaviours that were maintaining the depression side of things fell away...I found that as they were giving up smoking, there was a sense of, I guess … almost like a boost in their mood from giving up. The people I worked with were quite proud of themselves, what that did I think is increase their sense of control over their situation, I think they felt more empowered then to make other changes in their life, once they could see that actually they were giving up smoking, it almost opened up and they were like, “okay, if I can do that, then I can start working on maybe doing a little bit more exercise or looking at my sleep routine”, whatever it maybe that was in turn maintaining their depression symptoms." Southwest England service, female PWP (4 years’ experience), age 29 |
| **Challenges delivering the intervention** | Services are stretched for time | "I sometimes found it quite hard to squeeze in the smoking bit when someone was really quite, well, either low or anxious, as you only have half an hour appointments and it’s quite tight for time anyway. Southwest England service, female PWP (3 years’ experience), age 36 |
|  | Supporting clients with NRT | "I didn’t really know what was the best option...I think it’s quite a difficult thing to know... I think it would have been better if I’d had a step-by-step guide ... there was some information about them but not really like practicality, like when would you do what, what’s good for this, like what size patch do we give? Like it didn’t really give the recommendations which feels uncomfortable when I don’t really know anything about this and I don’t want to be giving someone something they don’t need, or not giving them enough... it just felt like a strange thing because I’m not a medical professional" Southwest England service, female PWP (2 years’ experience), age 27 |
| **Practical limitations of the trial** | Timing of trial recruitment | "... almost everybody that was smoking was saying no...so I'm thinking maybe something like somehow giving them some information around [smoking cessation] at some point, maybe when they do the referral or something like that, or maybe just advertising that we offer that so they could kind of have some idea before they do the initial assessment... It's a time issue because when we do, the initial assessment is just so much stuff we need to go through. But if we could maybe explain a little bit more about, you know why is it that we offer smoking cessation." London service, female PWP (5 years’ experience), age 35 |
|  | Logistical issues | "I guess because it was so long ago, I know when you guys came around to give us training, I’m the only person left, maybe actually [name], who’s my supervisor, still does it, but everyone else has left, and PWPs, there’s a high turnover of them, so there’s not that many other people within the service to go to, to say, ‘Is this the right thing?’ Southwest England service, female PWP (3 years’ experience), age 36 |

## Smoking cessation treatment-related feasibility outcomes

eTable 7 Average smoking cessation intervention duration in minutes. Data presented are mean duration in minutes with standard deviation (SD), and are presented by appointment, N=40.

|  | **Mean duration in minutes (SD)** | **N sessions that duration was reported by IAPT practitioners** |
| --- | --- | --- |
| Appointment 1 | 17.0 (8.3) | 40 |
| Appointment 2 | 13.1 (5.7) | 27 |
| Appointment 3 | 11.5 (5.8) | 24 |
| Appointment 4 | 9.8 (4.5) | 17 |
| Appointment 5 | 8.6 (4.9) | 16 |
| Appointment 6 | 10.0 (7.2) | 15 |
| Appointment 7 | 7.4 (4.1) | 11 |
| Appointment 8 | 6.2 (2.5) | 4 |
| Appointment 9 | 2.5 (3.5) | 2 |

eTable 8 Smoking cessation medicines/aids recommended to participants by IAPT practitioners at appointment 1. Data presented are frequency of medicine/aid type, with percent (%), N=37.

|  | **Frequency** | **%** |
| --- | --- | --- |
| No medication (i.e., cold turkey) | 5 | 13.5 |
| Single-nicotine replacement therapy (NRT) | 3 | 8.1 |
| Dual-NRT | 24 | 64.9 |
| Varenicline | 4 | 10.8 |
| E-cigarettes | 1 | 2.7 |

eTable 9 Smoking cessation medicines/aids recommended to participants by IAPT practitioners at appointment 2. Data presented are frequency of medicine/aid type, as percent (%), N=42.

|  | Frequency | % |
| --- | --- | --- |
| No medication (i.e., cold turkey) | 5 | 11.6 |
| Single-nicotine replacement therapy (NRT) | 4 | 9.3 |
| Dual-NRT | 28 | 65.1 |
| Varenicline | 2 | 4.7 |
| E-cigarettes | 4 | 9.3 |

## Blinded outcome data collection

eTable 10 Blinded researcher response to "At the very start of the telephone call - are you aware of which treatment arm the participant was allocated to?”. Data presented are answer frequency, as a percent (%) with numerator and denominator, at 3- and 6-month follow-up and by treatment arm.

|  | **3-months follow-up** | | **6-months follow-up** | |
| --- | --- | --- | --- | --- |
|  | **Frequency** | **%** | **Frequency** | **%** |
| Control |  |  |  |  |
| No | 38 | 84.4 | 45 | 84.9 |
| Yes | 7 | 15.6 | 8 | 15.1 |
| Treatment |  |  |  |  |
| No | 39 | 86.7 | 38 | 82.6 |
| Yes | 6 | 13.3 | 8 | 17.4 |

eTable 11 Blinded researcher response to "At the very start of the telephone call - are you aware of which treatment arm the participant was allocated to?”. Data presented are yes/no answer frequency, as a percent (%) with numerator and denominator, at 3- and 6-month follow-up and by Trust.

|  | **3-months follow-up** | | **6-months follow-up** | |
| --- | --- | --- | --- | --- |
|  | **Frequency** | **%** | **Frequency** | **%** |
| AWP |  |  |  |  |
| No | 10 | 47.6 | 15 | 62.5 |
| Yes | 11 | 52.4 | 9 | 37.5 |
| OX |  |  |  |  |
| No | 46 | 97.9 | 45 | 86.5 |
| Yes | 1 | 2.1 | 7 | 13.5 |
| NELFT |  |  |  |  |
| No | 16 | 100.0 | 16 | 100.0 |
| Yes | 0 | 0.0 | 0 | 0.0 |
| DIHCT |  |  |  |  |
| No | 5 | 83.3 | 7 | 100.0 |
| Yes | 1 | 16.7 | 0 | 0.0 |

## Number of days between randomisation and first IAPT appointment, and first IAPT appointment and 3-, and 6-months follow-up

eTable 12 Number of days between randomisation and first IAPT appointment, and first IAPT appointment and 3-, and 6-months follow-up. Data presented are mean differences and standard deviations, with p-values derived from two sample t-tests, N=135.

|  | **Control** | | **Treatment** | | **P-value from t-test** |
| --- | --- | --- | --- | --- | --- |
|  | **Mean difference** | **SD** | **Mean difference** | **SD** |  |
| Number of days between randomisation and 1^st^ IAPT appointment | 22.1 | 48.91 | 20.9 | 49.3 | 0.89 |
| Number of days between 1^st^ IAPT appointment and 3-months follow-up | 130.5 | 102.8 | 115.5 | 74.39 | 0.41 |
| Number of days between 1^st^ IAPT appointment and 6-months follow-up | 235.2 | 101.4 | 228.6 | 107.3 | 0.75 |

## Missing data monitoring for main trial outcomes from 2018 to 2022

eTable 13 Complete data for main trial outcomes at 3-month follow-up from 2020 to 2022. Data presented are percent % complete, with numerators and denominators.

| **Follow-up due date** | **N due** | **N with complete quit data at follow-up** | **% With complete quit data at follow-up** | **N with complete GAD-7 data at follow-up** | **% With complete GAD-7 data at follow-up** | **N with complete PHQ-9 data at follow-up** | **% With complete PHQ-9 data at follow-up** |
| --- | --- | --- | --- | --- | --- | --- | --- |
| October 2018 | 1 | 1 | 100.0% | 0 | 0.0% | 1 | 100.0% |
| November 2018 | 2 | 2 | 100.0% | 0 | 0.0% | 2 | 100.0% |
| December 2018 | 3 | 3 | 100.0% | 0 | 0.0% | 3 | 100.0% |
| January 2019 | 4 | 3 | 75.0% | 0 | 0.0% | 3 | 75.0% |
| March 2019 | 6 | 3 | 50.0% | 0 | 0.0% | 3 | 50.0% |
| May 2019 | 7 | 4 | 57.1% | 1 | 14.3% | 4 | 57.1% |
| June 2019 | 8 | 4 | 50.0% | 1 | 12.5% | 4 | 50.0% |
| July 2019 | 11 | 7 | 63.6% | 4 | 36.4% | 7 | 63.6% |
| August 2019 | 17 | 10 | 58.8% | 8 | 47.1% | 11 | 64.7% |
| September 2019 | 25 | 14 | 56.0% | 13 | 52.0% | 16 | 64.0% |
| October 2019 | 36 | 17 | 47.2% | 19 | 52.8% | 22 | 61.1% |
| November 2019 | 41 | 22 | 53.7% | 24 | 58.5% | 27 | 65.9% |
| December 2019 | 49 | 27 | 55.1% | 29 | 59.2% | 32 | 65.3% |
| January 2020 | 53 | 30 | 56.6% | 32 | 60.4% | 35 | 66.0% |
| February 2020 | 58 | 35 | 60.3% | 37 | 63.8% | 40 | 69.0% |
| March 2020 | 63 | 39 | 61.9% | 41 | 65.1% | 44 | 69.8% |
| April 2020 | 66 | 41 | 62.1% | 43 | 65.2% | 46 | 69.7% |
| May 2020 | 67 | 42 | 62.7% | 44 | 65.7% | 47 | 70.1% |
| June 2020 | 68 | 43 | 63.2% | 45 | 66.2% | 48 | 70.6% |
| September 2020 | 69 | 44 | 63.8% | 46 | 66.7% | 49 | 71.0% |
| October 2020 | 74 | 48 | 64.9% | 50 | 67.6% | 53 | 71.6% |
| November 2020 | 77 | 50 | 64.9% | 52 | 67.5% | 55 | 71.4% |
| December 2020 | 84 | 55 | 65.5% | 57 | 67.9% | 60 | 71.4% |
| January 2021 | 91 | 60 | 65.9% | 62 | 68.1% | 65 | 71.4% |
| February 2021 | 95 | 62 | 65.3% | 63 | 66.3% | 66 | 69.5% |
| March 2021 | 101 | 65 | 64.4% | 66 | 65.3% | 69 | 68.3% |
| April 2021 | 103 | 66 | 64.1% | 67 | 65.0% | 70 | 68.0% |
| May 2021 | 109 | 70 | 64.2% | 71 | 65.1% | 74 | 67.9% |
| June 2021 | 111 | 72 | 64.9% | 73 | 65.8% | 76 | 68.5% |
| July 2021 | 117 | 75 | 64.1% | 76 | 65.0% | 79 | 67.5% |
| August 2021 | 120 | 77 | 64.2% | 78 | 65.0% | 81 | 67.5% |
| September 2021 | 122 | 77 | 63.1% | 78 | 63.9% | 81 | 66.4% |
| October 2021 | 126 | 80 | 63.5% | 81 | 64.3% | 84 | 66.7% |
| December 2021 | 129 | 83 | 64.3% | 84 | 65.1% | 87 | 67.4% |
| January 2022 | 130 | 84 | 64.6% | 85 | 65.4% | 88 | 67.7% |
| March 2022 | 132 | 86 | 65.2% | 87 | 65.9% | 90 | 68.2% |
| May 2022 | 134 | 87 | 64.9% | 88 | 65.7% | 91 | 67.9% |
| June 2022 | 135 | 88 | 65.2% | 89 | 65.9% | 92 | 68.1% |

eTable 14 Complete data for main trial outcomes at 6-month follow-up from 2020 to 2022. Data presented are percent % complete, with numerators and denominators.

| **Follow-up due date** | **N due** | **N with complete quit data at follow-up** | **% With complete quit data at follow-up** | **N with complete GAD-7 data at follow-up** | **% With complete GAD-7 data at follow-up** | **N with complete PHQ-9 data at follow-up** | **% With complete PHQ-9 data at follow-up** |
| --- | --- | --- | --- | --- | --- | --- | --- |
| February 2019 | 1 | 1 | 100.0% | 1 | 100.0% | 1 | 100.0% |
| March 2019 | 3 | 1 | 33.3% | 1 | 33.3% | 1 | 33.3% |
| May 2019 | 4 | 2 | 50.0% | 2 | 50.0% | 2 | 50.0% |
| June 2019 | 5 | 2 | 40.0% | 2 | 40.0% | 2 | 40.0% |
| July 2019 | 6 | 2 | 33.3% | 2 | 33.3% | 2 | 33.3% |
| August 2019 | 7 | 3 | 42.9% | 3 | 42.9% | 3 | 42.9% |
| October 2019 | 9 | 4 | 44.4% | 4 | 44.4% | 4 | 44.4% |
| November 2019 | 11 | 6 | 54.5% | 6 | 54.5% | 6 | 54.5% |
| December 2019 | 15 | 8 | 53.3% | 9 | 60.0% | 9 | 60.0% |
| January 2020 | 24 | 15 | 62.5% | 15 | 62.5% | 15 | 62.5% |
| February 2020 | 34 | 22 | 64.7% | 22 | 64.7% | 22 | 64.7% |
| March 2020 | 38 | 26 | 68.4% | 26 | 68.4% | 26 | 68.4% |
| April 2020 | 47 | 32 | 68.1% | 32 | 68.1% | 32 | 68.1% |
| May 2020 | 51 | 35 | 68.6% | 35 | 68.6% | 35 | 68.6% |
| June 2020 | 57 | 40 | 70.2% | 40 | 70.2% | 40 | 70.2% |
| July 2020 | 63 | 44 | 69.8% | 44 | 69.8% | 44 | 69.8% |
| August 2020 | 65 | 46 | 70.8% | 46 | 70.8% | 46 | 70.8% |
| September 2020 | 67 | 48 | 71.6% | 48 | 71.6% | 48 | 71.6% |
| October 2020 | 68 | 49 | 72.1% | 49 | 72.1% | 49 | 72.1% |
| January 2021 | 71 | 49 | 69.0% | 49 | 69.0% | 49 | 69.0% |
| February 2021 | 75 | 52 | 69.3% | 52 | 69.3% | 52 | 69.3% |
| March 2021 | 82 | 55 | 67.1% | 55 | 67.1% | 55 | 67.1% |
| April 2021 | 87 | 57 | 65.5% | 57 | 65.5% | 57 | 65.5% |
| May 2021 | 93 | 61 | 65.6% | 61 | 65.6% | 61 | 65.6% |
| June 2021 | 96 | 63 | 65.6% | 63 | 65.6% | 63 | 65.6% |
| July 2021 | 102 | 66 | 64.7% | 66 | 64.7% | 66 | 64.7% |
| August 2021 | 103 | 67 | 65.0% | 67 | 65.0% | 67 | 65.0% |
| September 2021 | 109 | 70 | 64.2% | 70 | 64.2% | 70 | 64.2% |
| October 2021 | 112 | 73 | 65.2% | 73 | 65.2% | 73 | 65.2% |
| November 2021 | 116 | 76 | 65.5% | 76 | 65.5% | 76 | 65.5% |
| December 2021 | 121 | 79 | 65.3% | 79 | 65.3% | 79 | 65.3% |
| February 2022 | 125 | 82 | 65.6% | 82 | 65.6% | 82 | 65.6% |
| April 2022 | 128 | 85 | 66.4% | 85 | 66.4% | 85 | 66.4% |
| May 2022 | 129 | 86 | 66.7% | 86 | 66.7% | 86 | 66.7% |
| June 2022 | 130 | 86 | 66.2% | 86 | 66.2% | 86 | 66.2% |
| July 2022 | 132 | 88 | 66.7% | 88 | 66.7% | 88 | 66.7% |
| August 2022 | 133 | 88 | 66.2% | 88 | 66.2% | 88 | 66.2% |
| September 2022 | 134 | 89 | 66.4% | 89 | 66.4% | 89 | 66.4% |
| October 2022 | 135 | 90 | 66.7% | 90 | 66.7% | 90 | 66.7% |

eTable 15 Number of cases where researchers extracted PHQ-9, GAD-7, and smoking status data from IAPT clinical contact notes for participants who were lost to follow-up.

| **Follow-up** | **N PHQ-9 values imputed** | **N GAD-7 values imputed** | **N smoking cessation status imputed** |
| --- | --- | --- | --- |
| 3-months | 16 | 16 | 7 |
| 6-months | 3 | 3 | 0 |

eTable 16 Yearly drop out of trial as per patient request or by difficulty contacting. Data presented are number of participants and percent, by trial arm, N=135.

|  | | **Has the participant stated that they wish to stop providing follow-up data?** | | | | | **Has the follow-up not been completed due to difficulty contacting the participant?** | | | | |
| --- | --- | --- | --- | --- | --- | --- | --- | --- | --- | --- | --- |
| **Follow-up** | **Year of follow-up** | **Control** | | **Treatment** | | **% Difference between arms** | **Control** | | **Treatment** | | **% Difference between arms** |
|  |  | **N yes** | **% Yes** | **N yes** | **% Yes** |  | **N yes** | **% Yes** | **N yes** | **% Yes** |  |
| 3-months | 2018 | 0 | 0.0% | 0 | 0.0% | 0.0% | 0 | 0.0% | 0 | 0.0% | 0.0% |
|  | 2019 | 1 | 0.7% | 4 | 3.0% | 2.2% | 11 | 8.1% | 7 | 5.2% | -3.0% |
|  | 2020 | 0 | 0.0% | 3 | 2.2% | 2.2% | 4 | 3.0% | 2 | 1.5% | -1.5% |
|  | 2021 | 4 | 3.0% | 5 | 3.7% | 0.7% | 4 | 3.0% | 3 | 2.2% | -0.7% |
|  | 2022 | 0 | 0.0% | 0 | 0.0% | 0.0% | 0 | 0.0% | 1 | 0.7% | 0.7% |
| 6-months | 2019 | 1 | 0.7% | 3 | 2.2% | 1.5% | 3 | 2.2% | 3 | 2.2% | 0.0% |
|  | 2020 | 2 | 1.5% | 4 | 3.0% | 1.5% | 5 | 3.7% | 4 | 3.0% | -0.7% |
|  | 2021 | 3 | 2.2% | 7 | 5.2% | 3.0% | 2 | 1.5% | 4 | 3.0% | 1.5% |
|  | 2022 | 1 | 0.7% | 0 | 0.0% | -0.7% | 0 | 0.0% | 2 | 1.5% | 1.5% |

eTable 17 Overall drop out of trial as per patient request or by difficulty contacting. Data presented are number of participants and percent, by trial arm, N=135.

|  | **Has the participant stated that they wish to stop providing follow-up data?** | | | | | **Has the follow-up not been completed due to difficulty contacting the participant?** | | | | |
| --- | --- | --- | --- | --- | --- | --- | --- | --- | --- | --- |
|  | **Control** | | **Treatment** | | **% Difference between arms** | **Control** | | **Treatment** | | **% Difference between arms** |
|  | **N yes** | **% Yes** | **N yes** | **% Yes** |  | **N yes** | **% Yes** | **N yes** | **% Yes** |  |
| 3-months | 5 | 3.7% | 12 | 8.9% | 5.2% | 19 | 14.1% | 13 | 9.6% | -4.4% |
| 6-months | 7 | 5.2% | 14 | 10.4% | 5.2% | 10 | 7.4% | 13 | 9.6% | 2.2% |

# REFERENCES

1 Taylor GMJ, Aveyard P, Kessler D, *et al.* intEgrating Smoking Cessation treatment As part of usual Psychological care for dEpression and anxiety (ESCAPE): protocol for a randomised and controlled, multicentre, acceptability, feasibility and implementation trial. *Pilot Feasibility Stud* 2019. DOI:10.1186/s40814-018-0385-2.

2 Pentecost C, Farrand P, Greaves CJ, *et al.* Combining behavioural activation with physical activity promotion for adults with depression: findings of a parallel-group pilot randomised controlled trial (BAcPAc). *Trials 2015 161* 2015; **16**: 1–15.

3 Gierisch JM, Bastian LA, Calhoun PS, McDuffie JR, WillIAM-s JW. Smoking Cessation Interventions for Patients with Depression: A Systematic Review and Meta-analysis. *J Gen Intern Med* 2012; **27**: 351–60.

4 Royston P, White I. Multiple Imputation by Chained Equations (MICE): Implementation in *Stata*. *J Stat Softw* 2011. DOI:10.18637/jss.v045.i04.

5 Royston P. Multiple imputation of missing values. *Stata J* 2004; **4**: 227–41.
